# Attribution of Space‐Time Variability in Global‐Ocean Dissolved Inorganic Carbon

**DOI:** 10.1029/2021GB007162

**Published:** 2022-03-22

**Authors:** Dustin Carroll, Dimitris Menemenlis, Stephanie Dutkiewicz, Jonathan M. Lauderdale, Jess F. Adkins, Kevin W. Bowman, Holger Brix, Ian Fenty, Michelle M. Gierach, Chris Hill, Oliver Jahn, Peter Landschützer, Manfredi Manizza, Matt R. Mazloff, Charles E. Miller, David S. Schimel, Ariane Verdy, Daniel B. Whitt, Hong Zhang

**Affiliations:** ^1^ Moss Landing Marine Laboratories San José State University Moss Landing CA USA; ^2^ Jet Propulsion Laboratory California Institute of Technology Pasadena CA USA; ^3^ Department of Earth, Atmospheric and Planetary Sciences Massachusetts Institute of Technology Cambridge MA USA; ^4^ Center for Global Change Science Massachusetts Institute of Technology Cambridge MA USA; ^5^ Division of Geological and Planetary Sciences California Institute of Technology Pasadena CA USA; ^6^ Joint Institute for Regional Earth System Science and Engineering University of California Los Angeles Los Angeles CA USA; ^7^ Institute of Coastal Ocean Dynamics Helmholtz‐Zentrum Hereon Geesthacht Germany; ^8^ Max Planck Institute for Meteorology Hamburg Germany; ^9^ Scripps Institution of Oceanography University of California San Diego La Jolla CA USA; ^10^ NASA Ames Research Center Moffett Field CA USA

**Keywords:** carbon, ocean, sink, budget, model, equatorial

## Abstract

The inventory and variability of oceanic dissolved inorganic carbon (DIC) is driven by the interplay of physical, chemical, and biological processes. Quantifying the spatiotemporal variability of these drivers is crucial for a mechanistic understanding of the ocean carbon sink and its future trajectory. Here, we use the Estimating the Circulation and Climate of the Ocean‐Darwin ocean biogeochemistry state estimate to generate a global‐ocean, data‐constrained DIC budget and investigate how spatial and seasonal‐to‐interannual variability in three‐dimensional circulation, air‐sea CO_2_ flux, and biological processes have modulated the ocean sink for 1995–2018. Our results demonstrate substantial compensation between budget terms, resulting in distinct upper‐ocean carbon regimes. For example, boundary current regions have strong contributions from vertical diffusion while equatorial regions exhibit compensation between upwelling and biological processes. When integrated across the full ocean depth, the 24‐year DIC mass increase of 64 Pg C (2.7 Pg C year^−1^) primarily tracks the anthropogenic CO_2_ growth rate, with biological processes providing a small contribution of 2% (1.4 Pg C). In the upper 100 m, which stores roughly 13% (8.1 Pg C) of the global increase, we find that circulation provides the largest DIC gain (6.3 Pg C year^−1^) and biological processes are the largest loss (8.6 Pg C year^−1^). Interannual variability is dominated by vertical advection in equatorial regions, with the 1997–1998 El Niño‐Southern Oscillation causing the largest year‐to‐year change in upper‐ocean DIC (2.1 Pg C). Our results provide a novel, data‐constrained framework for an improved mechanistic understanding of natural and anthropogenic perturbations to the ocean sink.

## Key Points

- We evaluate the global dissolved inorganic carbon (DIC) budget for 1995–2018 using an ocean biogeochemistry state estimate Estimating the Circulation and Climate of the Ocean‐Darwin
- In the upper ocean, circulation provides the largest gain of DIC and biological processes are the dominant loss
- Interannual variability is greatest in equatorial regions and is associated with El Niño‐Southern Oscillation

## 1 Introduction

Since the outset of the industrial era, human activities such as fossil fuel combustion and cement production have rapidly increased the concentration of atmospheric carbon dioxide (CO_2_). On timescales relevant to this anthropogenic perturbation, the ocean constitutes the largest non‐geological carbon reservoir on Earth (37,200 ± 195 Pg C of DIC; Keppler et al., 2020), storing roughly 17 and 50 times more carbon than the terrestrial biosphere and pre‐industrial atmosphere, respectively (Friedlingstein et al., 2019). As such, the ocean plays a critical role in regulating global climate (Archer et al., 2009) and mitigating anthropogenic warming by sequestering atmospheric CO_2_ (i.e., the “ocean carbon sink”; Heinze et al., 2015). To date, the ocean sink has absorbed roughly 40% of industrial‐era fossil carbon emissions (Khatiwala et al., 2009, 2013; McKinley et al., 2017). This has motivated numerous studies to monitor patterns and trends in the ocean sink, with recent global estimates of anthropogenic carbon uptake yielding values of roughly 2.3 ± 0.6 (2000–2006; Khatiwala et al., 2009) and 2.6 ± 0.3 (1994–2007; Gruber et al., 2019) Pg C year^−1^. A major outstanding question is whether the oceans will continue to absorb roughly 40% of anthropogenic CO_2_ emissions in the future, or whether this fraction will change significantly as emissions and/or the climate state change.

The trajectory of the ocean sink is driven by processes that drive CO_2_ undersaturation (ocean uptake) and oversaturation (ocean outgassing) in surface waters. Many studies suggest an increase in the ocean sink during recent decades (Sarmiento & Gruber, 2002; Khatiwala et al., 2013; DeVries, 2014; DeVries et al., 2017, 2019); however, storage rates are subject to considerable variability over a range of space‐time scales. This hinders our ability to predict the long‐term variability and strength of the ocean sink (Landschützer et al., 2016; McKinley et al., 2020; Randerson et al., 2015) and limits efforts that inform policy in response to anthropogenic climate change (Dunne, 2016). Quantifying the magnitude and space‐time variability of the ocean sink has been recognized as an important goal in the Fifth Assessment Report of the Intergovernmental Panel on Climate Change (IPCC, 2013). In this study, we specifically focus on DIC as it: (a) is the sum of aqueous inorganic carbon species and represents ∼98% of the carbon in the ocean (Hansell, 2013) and (b) is directly linked to atmospheric CO_2_ concentrations.

Trends and patterns in the ocean carbon reservoir, specifically the DIC pool, are driven by the complex interplay of physical, chemical, and biological processes. Diagnosing and quantifying the space‐time variability of these drivers (Lauderdale et al., 2016; Lovenduski et al., 2013) is critical for a mechanistic understanding of the ocean DIC state and the future trajectory of its sources (DIC gain) and sinks (DIC loss). At the ocean surface, DIC concentration is modulated by ocean‐atmosphere CO_2_ exchange, which depends on CO_2_ solubility, air‐sea gas transfer velocity, and the difference between atmospheric and surface‐ocean partial pressure of CO_2_ (apCO_2_ and pCO_2_, respectively). Additionally, natural and anthropogenic vertical DIC gradients in the water column exhibit opposing signs (i.e., natural DIC typically increases with depth while anthropogenic DIC decreases with depth).

Three‐dimensional ocean circulation and mixing (DeVries et al., 2019; Silvano, 2020) subducts surface‐ocean DIC to depth, which isolates it from the atmosphere, and also upwells deep remineralized DIC back to the surface. This ventilated DIC is further transformed by exchange with the atmosphere and upper and intermediate waters, eventually being subducted back to depth (Talley, 2013). Biological processes redistribute DIC by uptake and fixation, export of particulates, remineralization of dissolved and particulate organic matter, dissolution of particulate inorganic carbon, and sedimentation of particulate matter — collectively termed the “biological pump” (Boyd et al., 2019).

To better characterize the ocean sink response to natural and forced variability, studies have used statistical extrapolation of surface‐ocean pCO_2_ and gas transfer parameterizations (e.g., Wanninkhof, 1992) to reconstruct maps of air‐sea CO_2_ flux and its space‐time variability (Landschützer et al., 2013; Rödenbeck et al., 2013, 2015; Takahashi et al., 2002; Wanninkhof et al., 2013). Most of these extrapolations leverage statistical relationships between in‐situ surface‐ocean pCO_2_, remotely sensed sea‐surface temperature (SST) and Chlorophyll (Chl), and climatological mixed‐layer depth (MLD) and sea‐surface salinity (SSS). Recent efforts have further extended these methods to monthly climatological DIC (Keppler et al., 2020) and surface‐ocean carbonate chemistry parameters (Gregor & Gruber, 2021). However, these extrapolations suffer from a number of methodological uncertainties, data sparsity, and observation noise (Fay et al., 2014). For example, since the mid‐1980s, less than 2% of all monthly 1° × 1° Surface Ocean CO_2_ Atlas (SOCAT; Bakker et al., 2016) grid cells contain surface‐ocean pCO_2_ observations, leading to diverging results in data‐sparse regions (Gloege et al., 2021; Ritter et al., 2017).

In contrast to extrapolation‐based methods, Ocean Biogeochemistry Models (OBMs; Aumont et al., 2015; Stock et al., 2014) have the ability to resolve the spatiotemporal scales necessary for full attribution of ocean sink variability. OBMs, however, do not typically assimilate physical and biogeochemical observations, which can lead to substantial misfit between the model solution and observations. Until very recently, studies that use data‐assimilative OBMs have either been (a) limited to regional studies, for example, the Southern Ocean (Rosso et al., 2017; Verdy & Mazloff, 2017), or (b) had global‐ocean coverage but a short assimilation window (e.g., 2009–2011 in Brix et al., 2015). With the advent of CO_2_‐dedicated satellites (e.g., the Observing Carbon Observatory 2 (OCO‐2); Crisp et al., 2017) and global carbon‐cycle data assimilation systems (e.g., the NASA Carbon Monitoring System Flux (CMS‐Flux) project; Ott et al., 2015; Bowman et al., 2017; Liu et al., 2014, 2017, 2021), there is a clear need for global‐ocean biogeochemical state estimates that accurately simulate regional patterns in the ocean sink over seasonal to multi‐decadal timescales. In fact, OCO‐2 observations enabled quantification of spatiotemporal changes in ocean‐atmosphere carbon exchange during the 2015–2016 El Niño event (Chatterjee et al., 2017; Patra et al., 2017). Additionally, top‐down CMS‐Flux estimates have recently been used to inform terrestrial ecosystem dynamics (Bloom et al., 2020; Quetin et al., 2020), which show potential for future applications to ocean biogeochemistry.

Here we use the Estimating the Circulation and Climate of the Ocean‐Darwin (ECCO‐Darwin) global‐ocean biogeochemistry state estimate (Carroll et al., 2020) to generate a data‐constrained DIC budget and investigate how spatiotemporal variability in advection and mixing, air‐sea CO_2_ flux, and the biological pump have modulated the ocean sink for 1995–2018. This study considers the sum of natural and anthropogenic DIC without, for the moment, separating the two contributions explicitly. ECCO‐Darwin assimilates physical (both ocean and sea‐ice) and biogeochemical observations, which greatly improves the model's fit to a suite of observations. To our knowledge, this is the first in‐depth analysis of the global‐ocean DIC budget using an ocean biogeochemistry state estimate. Our results provide novel three‐dimensional insight into the bio‐chemical‐physical decomposition of the DIC pool and trajectory of the ocean sink, priors for global carbon‐cycle inversions, and an improved framework for understanding sparse in‐situ biogeochemical observations.

## 2 Methods

### 2.1 ECCO‐Darwin: Physical State Estimate

The ECCO‐Darwin ocean biogeochemistry state estimate is extensively described in Brix et al. (2015), Manizza et al. (2019), and Carroll et al. (2020). The latest ECCO‐Darwin solution (version 5) is based on ocean circulation and physical tracers (temperature, salinity, and sea ice) from the Estimating the Circulation and Climate of the Ocean (ECCO) LLC270 global‐ocean and sea‐ice data synthesis (Zhang et al., 2018). ECCO LLC270 has nominal 1/3° horizontal grid spacing (∼18 km at high latitudes) and 50 vertical levels. Since the horizontal discretization is insufficient to resolve mesoscale eddies, their impact on large‐scale ocean circulation is parameterized using the Redi (1982) and Gent and McWilliams (1990) schemes; vertical mixing is parameterized with the Gaspar et al. (1990) scheme.

Physical observations are assimilated using the adjoint method (i.e., 4‐D‐Var; Wunsch et al., 2009; Wunsch & Heimbach, 2013), which minimizes a weighted least squares sum of model‐data misfit (the cost function) to optimize initial conditions, time‐varying surface‐ocean boundary conditions, and time‐invariant, three‐dimensional mixing coefficients for along‐isopycnal, cross‐isopycnal, and isopycnal thickness diffusivity. Because the initial conditions, surface boundary conditions, and mixing coefficients are estimated as part of the adjoint‐method optimization, the ECCO LLC270 ocean circulation estimate has negligible drift and therefore does not require spin‐up. The LLC270 circulation estimate is used at each time step (1200 s) to drive an online biogeochemistry and ecosystem model provided by the Massachusetts Institute of Technology Darwin Project (Dutkiewicz et al., 2015, 2020; Follows et al., 2007) – taken together these components form ECCO‐Darwin.

### 2.2 ECCO‐Darwin: Biogeochemical State Estimate

The Darwin model includes the cycling of carbon, nitrogen, phosphorus (PO_4_), iron (Fe), silica (SiO_2_), oxygen, and alkalinity. Matter is cycled from inorganic nutrients, through living and dead organic matter, and remineralized back to inorganic forms. All particulates are instantly removed once they reach the seafloor as a crude parameterization of sedimentation and to prevent model instability due to the accumulation of particulates at the bottom. Nitrogen cycling explicitly includes nitrate (NO_3_), nitrite (NO_2_), and ammonium pools (NH_4_). Carbonate chemistry is based on the efficient solver of Follows et al. (2006). Air‐sea CO_2_ flux is computed using the parameterization of Wanninkhof (1992) and forced with apCO_2_ from the zonally averaged National Oceanic and Atmospheric Administration Marine Boundary Layer Reference (NOAA MBL) product (Andrews et al., 2014). Atmospheric iron dust deposition at the ocean surface and terrestrial runoff along coastal boundaries is forced using the monthly climatology of Mahowald et al. (2009) and Fekete et al. (2002), respectively. Terrestrial runoff consists of freshwater only and does not include nutrients nor DIC.

The Darwin ecology includes five large‐to‐small phytoplankton functional types (diatoms, other large eukaryotes, *Synechococcus*, and low‐ and high‐light adapted *Prochlorococcus*), along with two zooplankton types that graze preferentially on either large eukaryotes or small picoplankton. Biogeochemical properties (i.e., inorganic carbon and nutrients, phytoplankton and zooplankton, dissolved organic matter, and detrital particles) are treated as prognostic variables and are advected and mixed by the LLC270 ocean circulation.

The biogeochemical model is optimized separately from the circulation using a low‐dimensional Green's Functions approach (Menemenlis et al., 2005) to assimilate a variety of biogeochemical observations and to adjust the Darwin initial conditions and six ecological parameters. The mixing coefficients from the adjoint optimization are applied to both the physical and biogeochemical fields. A detailed description of the ECCO‐Darwin model setup, observational constraints, and optimization methodology is presented in Carroll et al. (2020).

### 2.3 Model Bias and Drift

**Figure 1:**
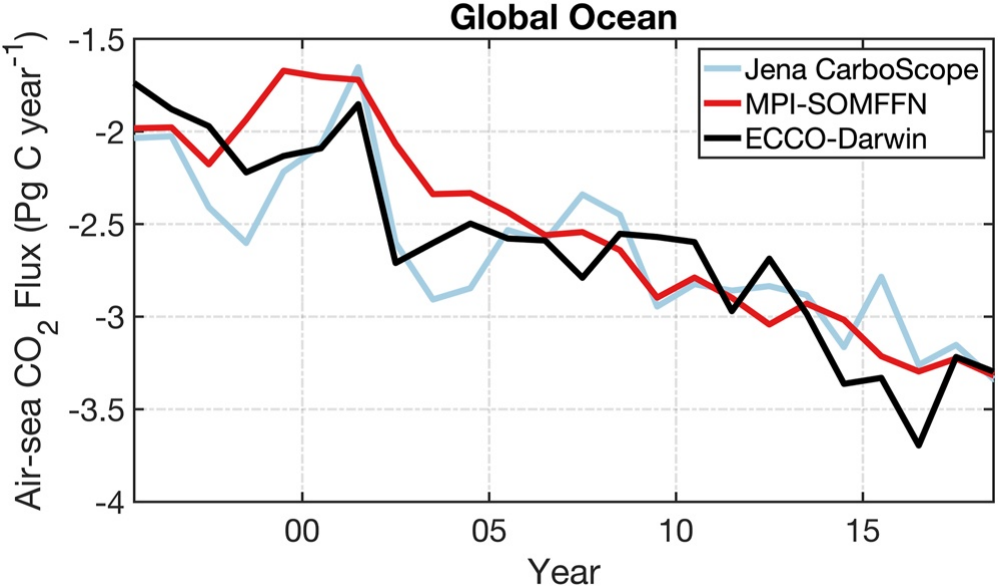
Globally integrated air‐sea CO_2_ flux time series for Jena CarboScope v2021 (light blue line), MPI‐SOMFFN v2021 (red line), and Estimating the Circulation and Climate of the Ocean‐Darwin (black line). Air‐sea CO_2_ fluxes are annual means; all values represent ocean uptake. Note that a runoff contribution from air‐sea CO_2_ flux of 0.78 Pg C year^−1^ (Resplandy et al., 2018) has been subtracted from Jena Carboscope and MPI‐SOMFFN, as done in Le Quéré et al. (2018).

An important consideration for the present study is that of model bias and drift. A fully spun‐up model will tend to have large bias but negligible drift. Conversely, a model that is initialized close to observations will tend to have small bias but large drift. For the present study, we aim for a compromise between bias and drift that reduces large upper‐ocean initialization transients while maintaining the model DIC concentration close to observed profiles in the deep ocean. As shown in Brix et al. (2015), the Green's function approach can simultaneously reduce both bias and drift for biogeochemistry, similar to the adjoint method used for the circulation. Because the Green's Function approach is low dimensional (only a small number of model parameters can be adjusted), some residual model drift is removed by discarding the first 3 years (1992–1994) of the ECCO‐Darwin simulation in our analysis. Figure 1 shows that the globally integrated ECCO‐Darwin air‐sea CO_2_ flux is consistent with the Jena CarboScope (Rödenbeck et al., 2013) and MPI‐SOMFFN (Landschützer et al., 2016) products. The ECCO‐Darwin ocean carbon sink trend matches that of the two observation‐based estimates, indicating that the ECCO‐Darwin global‐ocean CO_2_ flux has minimal model drift during the 1995–2018 model period. A more detailed region‐by‐region discussion of ECCO‐Darwin CO_2_ flux bias and drift is provided in Carroll et al. (2020).

### 2.4 DIC Budget

**Figure 2:**
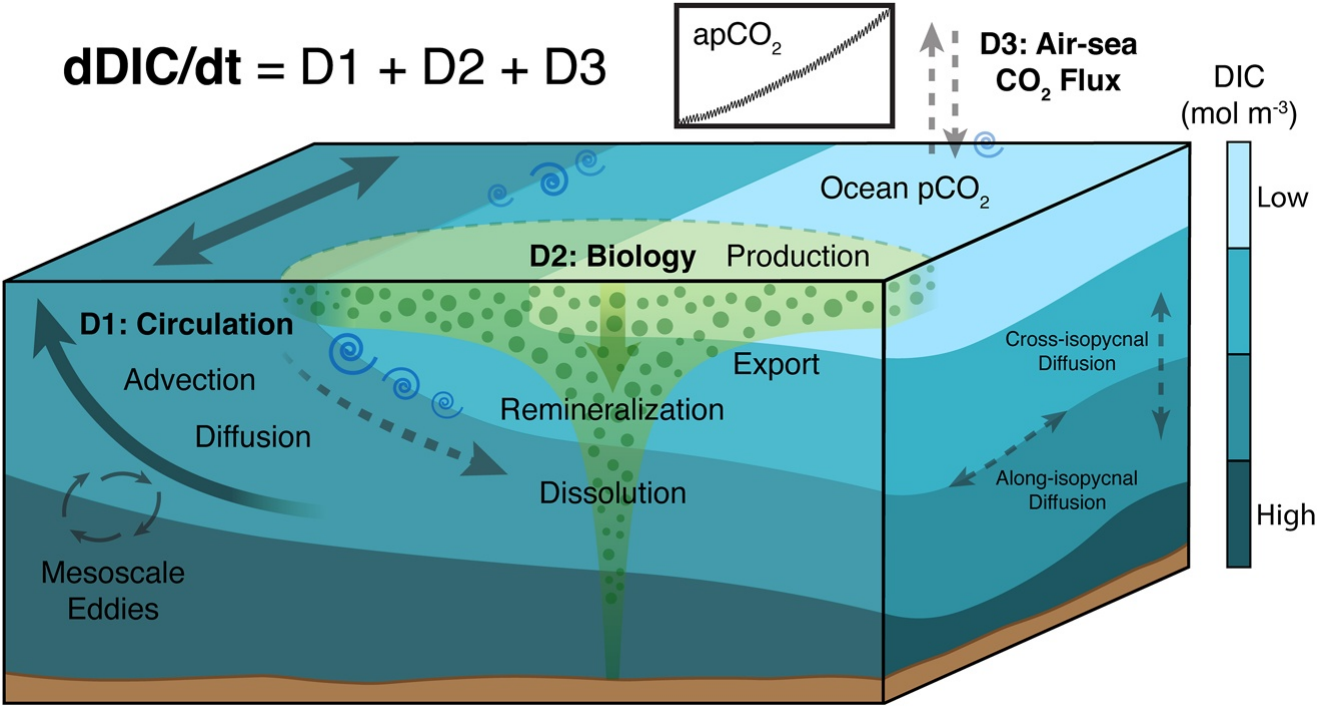
Schematic of Estimating the Circulation and Climate of the Ocean‐Darwin DIC budget terms. Budget term **D1** represents ocean circulation, with contributions from divergence of circulation and mixing (∇⋅*DIC*_*Adv*_ and ∇⋅*DIC*_*Dif*_ in Equation 1, respectively). ∇⋅*DIC*_*Adv*_ includes transport due to parameterized mesoscale eddies and ∇⋅*DIC*_*Dif*_ includes along‐ and cross‐isopycnal diffusion. Terms **D2** and **D3** show biological processes and air‐sea CO_2_ flux, respectively (*DIC*_*Bio*_ and *F*_*CO2*_ in Equation 1). DIC increases with depth (light to dark blue colors) as particulate carbon is exported to depth and is remineralized and dissolved from acidification. A further decomposition of *DIC*_*Bio*_ is given in Equation 2.

A key strength of ECCO‐Darwin is that all physical and biogeochemical fields are conserved within numerical precision over the entire model period. Therefore, ECCO‐Darwin exactly obeys conservation laws and there are no spurious sources and sinks of volume, heat, salt, carbon, etc. This allows for the analysis of a data‐constrained, fully closed DIC budget that permits attribution to ocean circulation, air‐sea CO_2_ flux, and biological processes (Figure 2).

In the open ocean (i.e., neglecting river and land sources of carbon), the time‐evolution of DIC is described by the three‐dimensional budget equation,

(1) $$\frac{dDIC}{dt}=-\nabla \cdot DIC_{Adv}-\nabla \cdot DIC_{Dif}+DIC_{Bio}+F_{CO_{2}}.$$

here ∇⋅*DIC*_*Adv*_ and ∇⋅*DIC*_*Dif*_ represent the divergence of horizontal and vertical advective DIC transport and diffusive processes, respectively (Figure 2, **D1**). DIC_Bio_ represents biological processes (Figure 2, **D2**) and ${F_{CO}}_{2}$ is the contribution of air‐sea CO_2_ flux to the DIC tendency (Figure 2, **D3**).

*DIC*_*Adv*_ includes the parameterization of transport due to mesoscale eddies from Gent et al. (1995). The diffusion term *DIC*_*Dif*_ represents unresolved flows and eddies, for example, convective adjustment, along‐isopycnal diffusion from the Redi (1982) scheme, and diapycnal and mixing layer diffusion from the Gaspar et al. (1990) scheme.

Biological processes can be further decomposed as

(2) $$DIC_{Bio}=Org_{Prod}+PIC_{Prod}+PIC_{Diss}+Org_{Remin},$$

where *Org*_*Prod*_ and *PIC*_*Prod*_ refer to the biological uptake of DIC for production of organic matter and particulate inorganic matter, respectively. Particulate inorganic matter comes from calcification and is referred to as PIC. *PIC*_*Diss*_ is the dissolution of PIC and *Org*_*Remin*_ is remineralization of organic matter.

We compute the monthly mean DIC budget in each grid cell during January 1995 to December 2018. The three‐dimensional DIC budget is then volume‐integrated over the upper 100 m and over the full‐depth water column, yielding a mass budget. The 100‐m integration depth was chosen because it is similar to the global‐mean winter wind‐driven mixed layer depth, euphotic zone depth, and is a conventional reference depth for export calculations; potential caveats and sensitivities to this choice will be discussed.

In the ECCO‐Darwin model configuration, the injection or removal of surface‐ocean freshwater does not impact DIC mass directly, even though it changes surface DIC concentration. The addition or removal of surface freshwater due to precipitation, evaporation, and terrestrial runoff has two consequences. The first is a surface pressure perturbation (or volume change) that is instantaneously distributed globally. The second is a transport of DIC‐rich ocean waters away from or toward the location of the surface flux; this effect is captured by the ∇ ⋅ *DIC*_*Adv*_ term.

Because exchanges between sea ice and the ocean do not add or remove mass locally, freshwater fluxes due to sea‐ice melt and freeze do not transport DIC‐rich ocean waters away from or toward the location of the flux. Nevertheless, because the ECCO‐Darwin simulation uses the z* rescaled vertical coordinates of Campin et al. (2008), dilution or concentration of DIC due to sea‐ice melting or freezing is still, to first order, captured by ∇ ⋅ *DIC*_*Adv*_ (Text S1 in Supporting Information S1).

For the remainder of the text, we use the following terminology to represent the DIC budget terms: $\frac{dDIC}{dt}$ = DIC tendency, ∇ ⋅ *DIC*_*Adv*_ = net advection, ∇ *DIC*_*Dif*_ = net diffusion, F_CO2_ = air‐sea CO_2_ flux, and *DIC*_*Bio*_ = net biology. For the further decomposition of net biology: Org_Prod_ = organic production, PIC_Prod_ = PIC production, *PIC*_*Diss*_ = PIC dissolution, and *Org*_*Remin*_ = organic remineralization. A summary of all budget terms is shown below in Table 1.

**Table 1:** Summary of DIC Budget Terms

**DIC budget term**	**Description**
$\frac{dDIC}{dt}$	DIC tendency
∇ ⋅ *DIC*_Adv_	Net advection
∇ ⋅ *DIC*_*Dif*_	Net diffusion
F_CO2_	Air‐sea CO_2_ flux
*DIC*_*Bio*_	Net biology
Org_Prod_	Organic production
PIC_Prod_	PIC production
PIC_Diss_	PIC dissolution
Org_Remin_	Organic remineralization

### 2.5 Biome‐Scale Analysis

**Figure 3:**
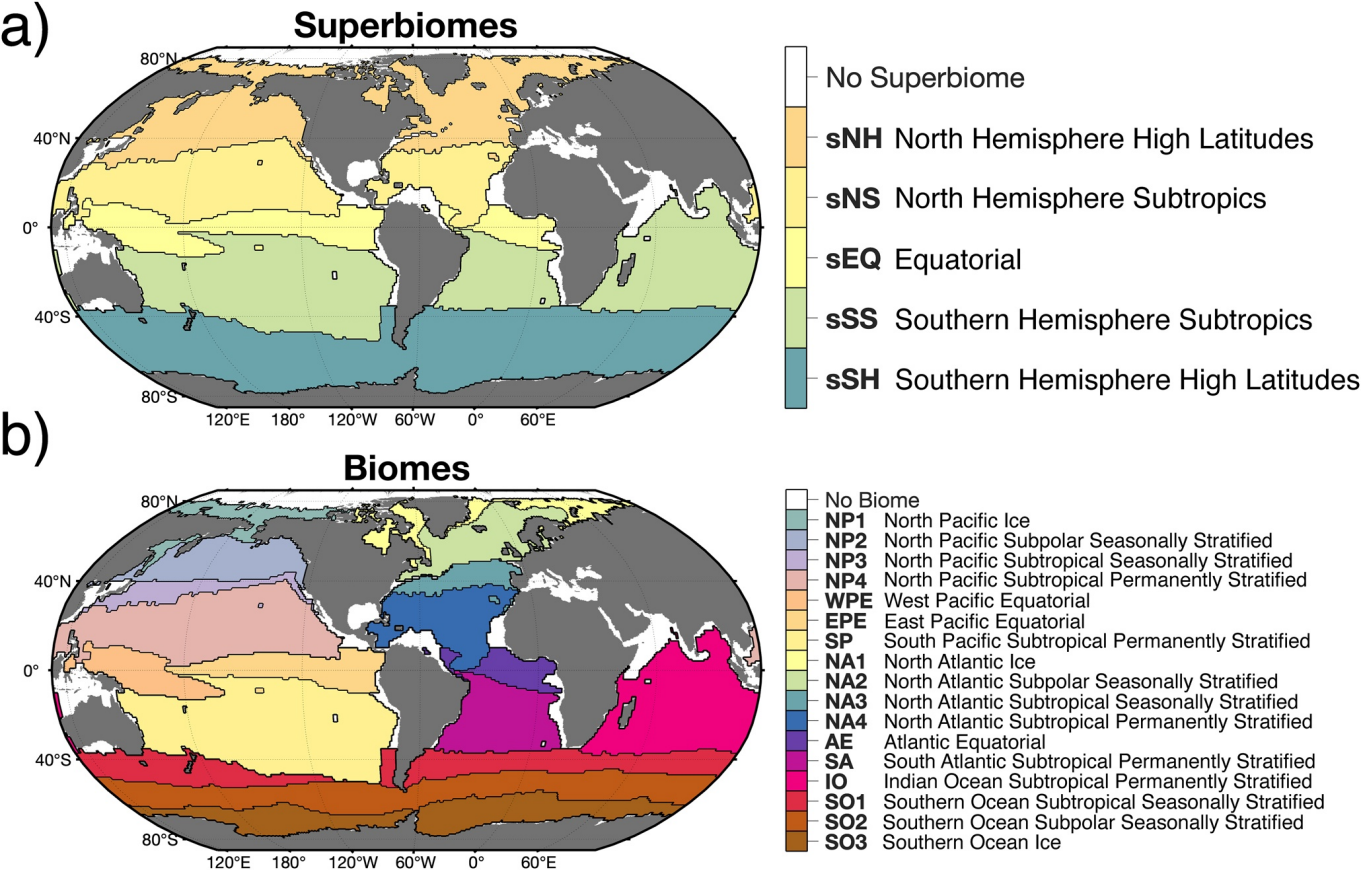
(a) Superbiome and (b) biome regions adapted from Fay and McKinley (2014). Biomes are determined from coherence between monthly SST, spring and summer Chl, sea‐ice fraction, and climatological maximum MLD. White colors indicate regions that do not fit the criteria for any biome and hence are excluded from our analysis. The superbiomes in (a) are constructed by combining the individual biomes in (b). **sNH** = NP1 + NP2 + NP3 + NA1 + NA2 + NA3; **sNS** = NP4 + NA4; **sEQ** = WPE + EPE + AE; **sSS** = SP + SA + IO; **sSH** = SO1 + SO2 + SO3.

We compute time series of the DIC budget terms in the time‐invariant, open‐ocean biome regions of Fay and McKinley (2014). The 5 superbiomes (Figure 3a) and 17 biomes (Figure 3b) provide nearly full coverage of the open ocean and are determined from coherence between monthly SST, spring and summer Chl, sea‐ice fraction, and climatological maximum MLD for 1998–2010. For simplicity, most of the figures and discussion in this paper pertain to the superbiomes, but we also provide similar analysis for all 17 biomes both in the manuscript and in the Supporting Information for additional detail. These biomes provide a more robust estimate of biogeochemical ocean provinces compared to regions that use arbitrary rectangular boundaries and are a natural choice for clustering carbon cycle dynamics. The biomes are mapped onto the ECCO‐Darwin grid using nearest‐neighbor interpolation. We note that each biome has a different surface area and hence a different mass‐budget volume. Therefore, we normalize by volume (and compute volume‐weighted means) when comparing budget terms across different biomes, resulting in a concentration budget.

## 3 Results

### 3.1 Comparison to Observations

**Figure 4:**
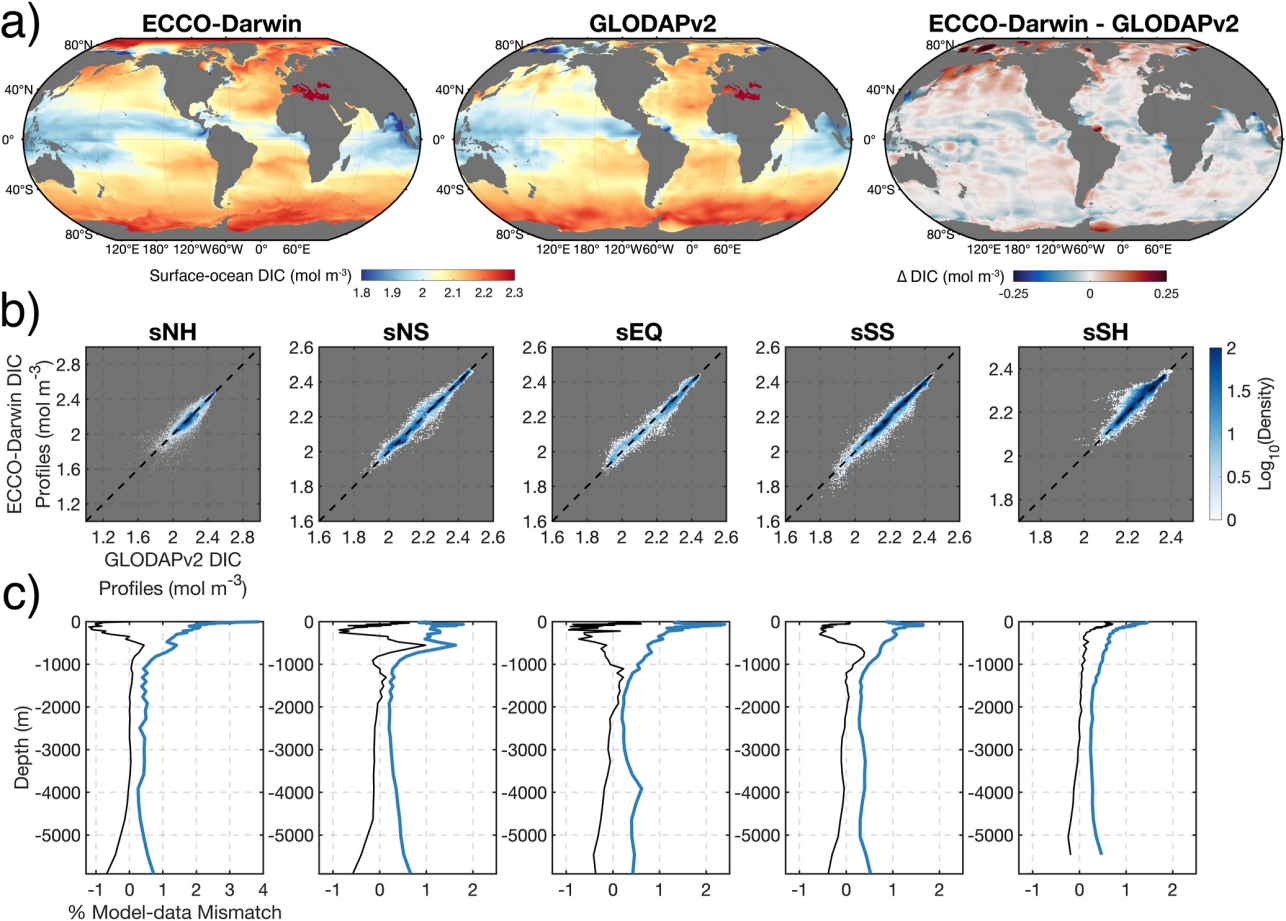
(a) Comparison of Estimating the Circulation and Climate of the Ocean (ECCO)‐Darwin climatological surface‐ocean dissolved inorganic carbon (DIC) with the GLODAPv2 mapped product (Lauvset et al., 2016). (b) Comparison of DIC vertical profiles from ECCO‐Darwin and in‐situ GLODAPv2.2021 observations (Olsen et al., 2020) in each superbiome. The *x*‐axis shows observations and *y*‐axis shows the corresponding monthly mean model value taken at the closest space‐time (x,y,z,t) location. Colors represent the density of model‐data pairs in log scale (i.e., blue colors = more observations available). The corresponding R^2^ values are shown in the upper left of each panel. (c) Area‐weighted vertical profiles of mean (thin black line) and standard deviation (thick blue line) model‐data difference. The mean difference and standard deviation are normalized by the mean observed value at each depth and reported in %.

We first benchmark ECCO‐Darwin climatological DIC (1995–2018) with the GLODAPv2 mapped product (Lauvset et al., 2016) and GLODAPv2.2021 in‐situ vertical profiles (Lauvset et al., 2021; Figure 4 and Figure S1 in Supporting Information S1). We note that the previous study of Carroll et al. (2020) extensively evaluated ECCO‐Darwin against SOCATv5 surface‐ocean pCO_2_ observations (Bakker et al., 2016); therefore, here we focus only on model‐data comparison of DIC. ECCO‐Darwin generally reproduces the observed latitudinal gradient in surface‐ocean DIC, along with the spatial patterns of low DIC near major river deltas and in the Equatorial, West Pacific, and Indian Oceans (Figure 4a). In the Greenland, Iceland, and Norwegian Seas, Irminger Sea, Labrador Sea, North Pacific Ocean, and the central Arctic Ocean (where GLODAPv2 lacks observations), ECCO‐Darwin generally exhibits higher surface‐ocean DIC concentrations compared to the GLODAPv2 mapped product.

ECCO‐Darwin vertical DIC profiles show good agreement with in‐situ GLODAPv2.2021 observations taken at the closest space‐time (*x*,*y*,*z*,*t*) location in each superbiome (Figure 4b). The model‐data pairs with the most available observations (blue colors) generally fall along the 1:1 line (Figure 4b, dashed black line), indicating that the model matches observations.

Examination of the mean (Figure 4c, thin black line) and standard deviation (Figure 4c, thick blue line) model‐data difference at each depth and in each superbiome reveals a very good fit between model and observations. A maximum standard‐deviation difference of 3.9% is reached in the surface level of sNH, resulting from mismatch in locations near sea ice (Figure 4b, see white outliers in sNH). For all other superbiomes, the maximum standard‐deviation difference in the upper 100 m is less than 2.4%. Over the 24‐year period of analysis, the majority of DIC variability occurs in the mixed layer and upper ocean, where convection and overturning circulation is vigorous. The standard‐deviation model‐data mismatch below the 1000‐m depth is less than 0.7% in all superbiomes.

### 3.2 Budget Climatology

#### 3.2.1 Climatological Fields

**Figure 5:**
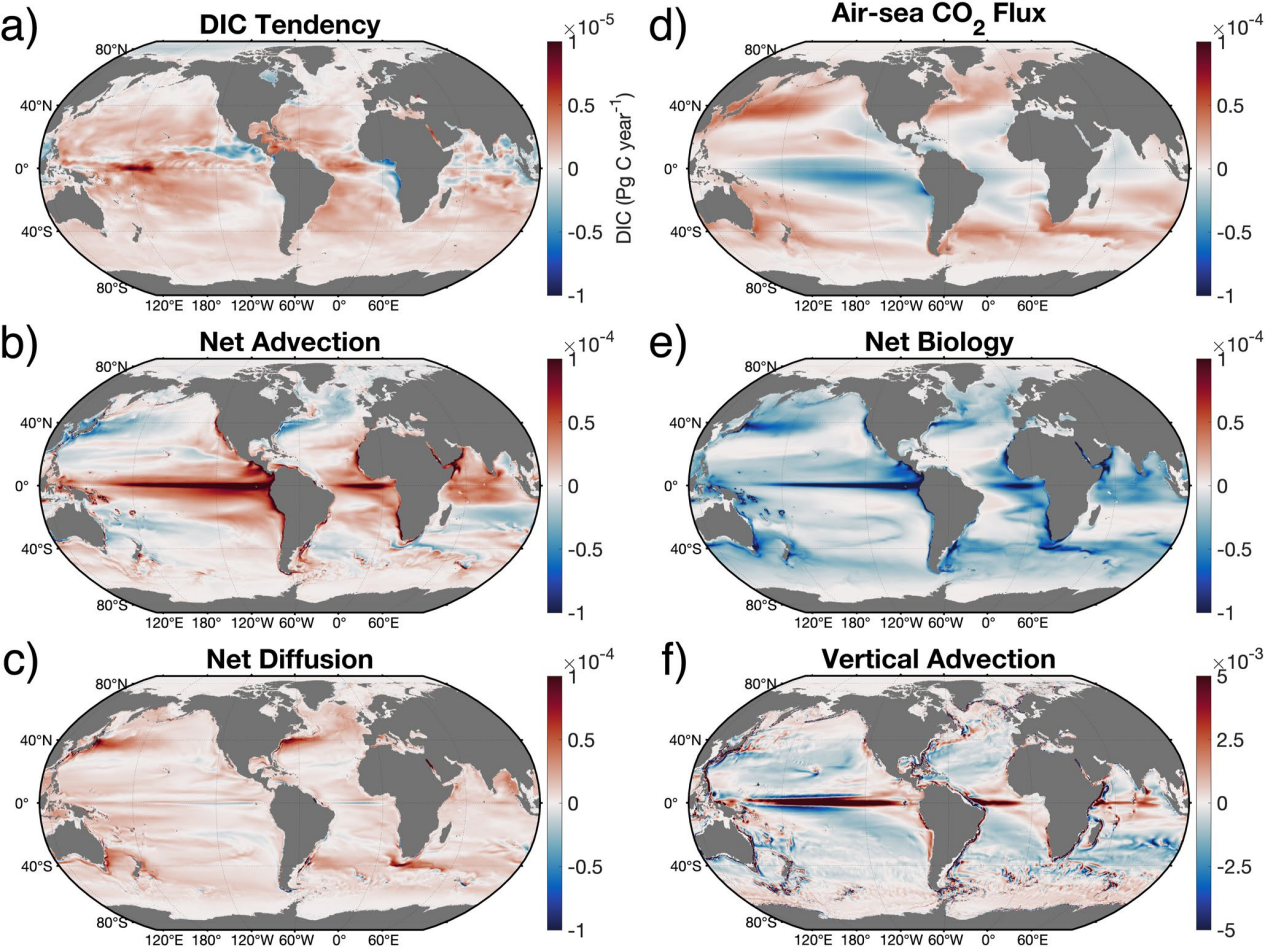
1995–2018 time‐mean climatology of mass budget terms, vertically integrated from the surface to 100‐m depth. Positive values (red colors) represent a gain of DIC and negative values (blue colors) show DIC loss. The DIC tendency shown in (a) is the sum of panels (b)**–**(e). Net advection (b) and net diffusion (c) are the sum of horizontal and vertical components. The vertical advection term is shown in (f) to highlight the role of equatorial upwelling (red colors). Note the different scales used in (a) and (f) relative to the other panels, indicating the strong compensation between the various budgets terms and between vertical and horizontal advection.

Having shown that ECCO‐Darwin can accurately simulate the surface‐ocean patterns and vertical structure of the observed DIC pool, we next examine climatological fields (January 1995 to December 2018) from the DIC mass budget in the upper 100 m (Figure 5). The DIC tendency (Figure 5a), which is the sum of the budget terms shown in Figures 5b–5e, exhibits a strong latitudinal gradient and shows a general increase of DIC in the tropics, subtropics, and Southern Ocean. The largest gains and losses of DIC occur in the equatorial ocean and along select coastal zones (e.g., the periphery of west Central America and West Africa), respectively. Globally, the largest DIC tendency magnitudes are found in the West Equatorial Pacific Ocean. The Arctic Ocean is a region of weak DIC loss, with negative DIC tendencies concentrated in the central Arctic and offshore of the Kara and Laptev marginal seas.

Net advection results in a gain of DIC in equatorial and coastal upwelling regions and a loss of DIC in the Kuroshio, Gulf Stream, and Agulhas Current systems and their respective extensions (Figure 5b). In upwelling regions, net advection results in a gain of DIC and is dominated by the vertical transport of deep, DIC‐rich waters (Figure 5f). In western boundary currents, net advection results in DIC loss, driven by the poleward transport of tropical, low‐DIC waters. The Southern Ocean is also a region of advective DIC gain, albeit weaker than the equatorial and tropical regions, due to the large‐scale upwelling (Ekman suction) of DIC‐rich waters (Figure 5f). Weaker and more diffuse advective losses of DIC due to downwelling (Ekman pumping) are found in the Northern and Southern Hemisphere subtropical gyres.

We note that the magnitude of the horizontal (not shown) and vertical advection terms (Figure 5f) are roughly an order of magnitude larger than net advection, demonstrating a substantial level of horizontal‐vertical compensation. For example, increased DIC due to horizontal convergence in the Pacific subtropical gyres (not shown) is partially compensated by the divergence of DIC from downwelling (Figure 5f, blue colors), leading to regions with weak DIC loss. Near the Equator, the vertical upwelling of DIC‐rich waters is not fully compensated by horizontal divergence from Ekman transport and poleward flow into the subtropical gyres, leading to a gain of DIC. Globally integrated, net advection provides a DIC gain of 2.9 Pg C year^−1^ to the upper 100 m.

Net diffusion generally provides a gain of DIC (Figure 5c) and is dominated by the vertical component due to large vertical DIC gradients in the upper 100 m. Net diffusion hotspots are primarily found in western boundary current systems, where low‐DIC waters of tropical origin mix with deeper DIC‐rich waters during winter months. At these hotspot locations, positive net diffusion exceeds negative net advection. Therefore, the sum of advection and diffusion, that is, ocean circulation, results in an overall gain of DIC. Globally integrated, net diffusion provides a DIC gain of 3.4 Pg C year^−1^. Locally, DIC loss due to diffusion is small and primarily pertains to horizontal diffusion of DIC‐rich waters away from upwelling regions. Together, advection and diffusion contribute 6.3 Pg C year^−1^ to the upper 100 m, which is to a large extent balanced by net biology.

Air‐sea CO_2_ flux (Figure 5d) drives CO_2_ outgassing (DIC loss) in equatorial regions and offshore of several coastal upwelling zones (e.g., western North America, Central and South America, and northwest Africa). The strongest open‐ocean CO_2_ outgassing region is located between 2°S and 8°S in the Eastern Equatorial Pacific Ocean. The most vigorous CO_2_ uptake (DIC gain) occurs in subpolar and western boundary current regions, where (a) pCO_2_ solubility (and hence CO_2_ uptake) increases as warm surface waters cool when flowing poleward and (b) strong biological fixation draws down DIC in the euphotic zone. Additionally, poleward‐flowing western boundary current waters gain anthropogenic CO_2_ as they enter the subpolar gyres, where upwelling exposes waters with low anthropogenic CO_2_ concentrations to the surface. In total, air‐sea CO_2_ flux provides a global‐ocean DIC gain of 2.6 Pg C year^−1^.

Net biology causes DIC loss in equatorial and high‐latitude regions, due to strong organic production that is not fully compensated by organic remineralization in the upper 100 m (Figure 5e and Figure S2 in Supporting Information S1). Near the Equator, increased organic production is driven primarily by the upwelling of nutrients (not shown) and consistent, year‐round insolation. In the oligotrophic subtropical gyres, the biological loss of DIC is weak. Globally integrated, net biology results in a DIC loss of 8.6 Pg C year^−1^, which has the largest magnitude of all budget terms. Locally, we note that DIC gain occurs where net transport drives convergence of organic material and shallow remineralization outstrips export. The residual between circulation, air‐sea CO_2_ flux, and net biology (i.e., the DIC tendency) results in a DIC gain of 0.3 Pg C year^−1^ in the upper 100 m.

#### 3.2.2 Climatological Zonal‐Mean Structure

**Figure 6:**
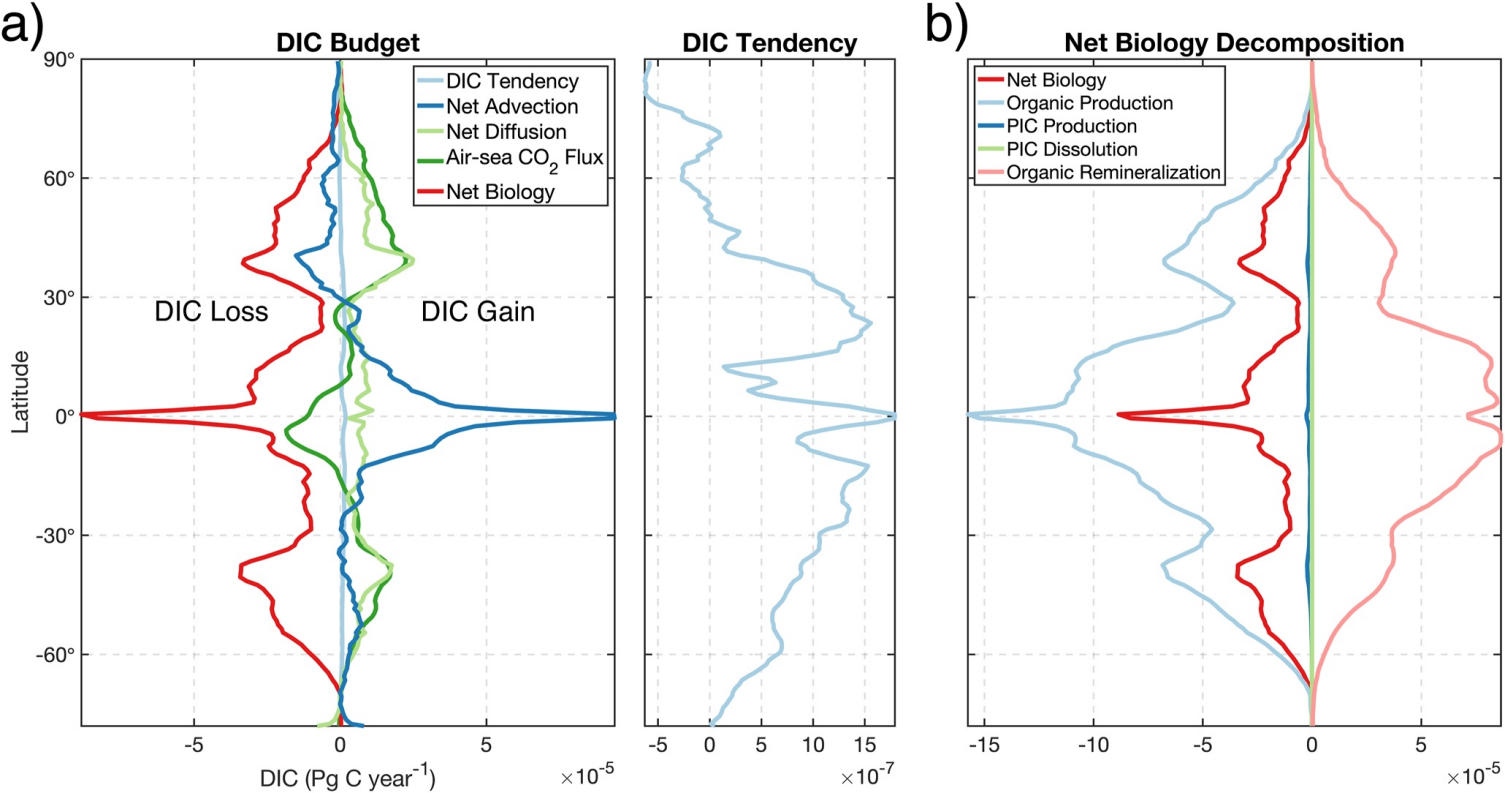
(a) 1995–2018 time‐mean climatology of mass budget terms shown as a function of latitude. A zoom‐in of the DIC tendency is shown on the right‐hand side of (a). Panel (b) shows a further decomposition of the net biology term, shown as a red line in (a). Values are vertically integrated from the surface to the 100‐m depth and then zonally averaged into 1° latitudinal bands. Positive values represent DIC gain and negative values are loss.

Examination of the zonally averaged mass budget as a function of latitude reveals the intricate pole‐to‐pole balance between budget terms (Figure 6). The DIC tendency is roughly an order of magnitude smaller than the other terms, demonstrating substantial compensation in the net budget (Figure 6a, light blue lines). The largest global magnitudes of net advection (dark blue line) and net biology (red line) are found in a narrow equatorial band between 10°S and 5°N, with equatorial upwelling of DIC‐rich waters outpacing biological production. Further north, from 5 to 72°N, this pattern reverses and net biology exceeds net advection. Both terms cause a loss of DIC from 30 to 79°N, highlighting the role of strong advective divergence and biological production in the Northern Hemisphere boundary‐current systems, Subpolar North Atlantic, and Nordic Seas. In the Southern Hemisphere, from 10 to 70°S, DIC loss from net biology dominates the DIC gain from net advection.

Near the Equator and tropics, net diffusion (light green line) is roughly 8× smaller than net advection. In subtropical and subpolar latitudes (30–60°N and 25–60°S), the magnitude of net diffusion is generally larger than net advection due to mode water formation and intense vertical mixing in boundary currents. These terms tend to oppose each other in the subpolar Northern Hemisphere, with DIC loss from advective divergence being overcompensated by DIC gain from net diffusion. However, in the subpolar Southern Hemisphere, where Southern Ocean upwelling is active, both net advection and net diffusion result in a gain of DIC. Air‐sea CO_2_ flux (dark green line) generally results in a loss of DIC (CO_2_ outgassing) in the equatorial and tropical regions and a gain of DIC (CO_2_ uptake) in the subtropics, subpolar regions, and Southern Ocean.

Decomposition of the net biology term (Figure 6b, red line) demonstrates that organic production (light blue line) is partially compensated by organic remineralization (light pink line) at all latitudes in the upper 100 m. The magnitude of both terms is reduced around 30°N and 30°S, where the oligotrophic conditions of the subtropical gyres limit biological productivity and export. PIC production (dark blue line) is roughly two orders of magnitude smaller than organic production, with PIC dissolution (light green line) providing a very small global‐ocean gain of DIC.

### 3.3 Budget Seasonal Climatology

#### 3.3.1 Seasonal Zonal‐Mean Structure

**Figure 7:**
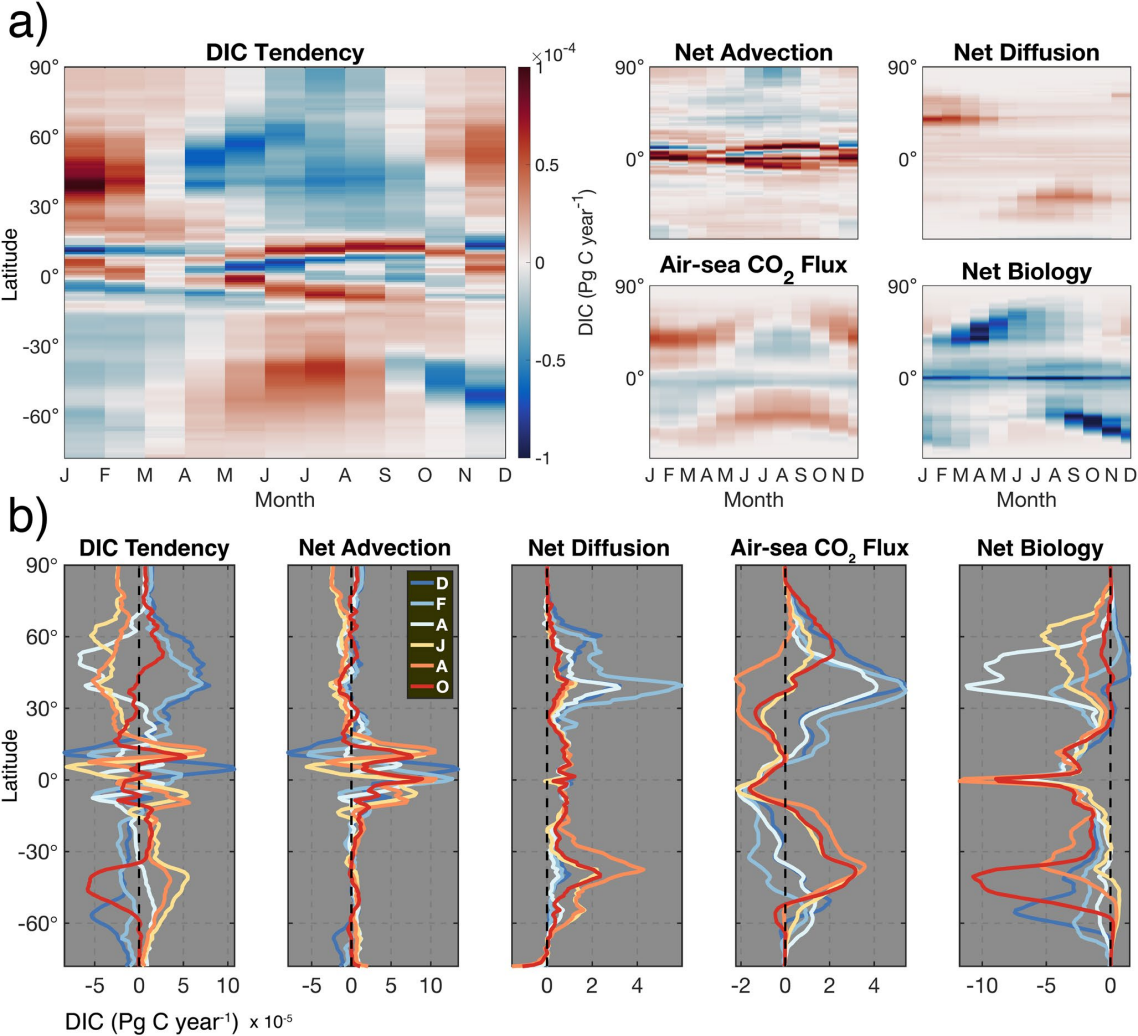
(a) Seasonal climatology of mass budget terms shown as a function of latitude; values are vertically integrated from the surface to the 100‐m depth and then zonally averaged into 1° latitudinal bands. (b) Line plot of budget terms showing select months (December, February, April, June, August, and October); dashed black line shows the zero value. Note the different *x*‐axis scales used in (b).

We next examine the seasonal climatology of budget terms as a function of latitude (Figure 7). DIC tendency exhibits clear seasonality in both hemispheres, with a stronger seasonal cycle in the Northern Hemisphere. Throughout the equatorial band, intricate patterns in net advection, which shift latitudinally throughout the year, drive the largest advective seasonal cycle in the global ocean. In the Arctic Ocean and periphery of Antarctica, the formation and melt of sea ice causes advective divergence and convergence of DIC, respectively; this effect is strongest in the Arctic Ocean during boreal summer. Seasonality in net diffusion results from increased vertical mixing during winter and spring. This occurs primarily in boundary current regions and throughout mode water formation regions in the Northern North Atlantic and Southern Ocean (Figure 7b). This is due to intense vertical mixing in the Gulf Stream, Kuroshio, Agulhas, and East Australian Current systems.

Both air‐sea CO_2_ flux and net biology have a seasonal structure that strongly varies with latitude. For air‐sea CO_2_ flux, this latitudinal shift is driven by a combination of physical and biogeochemical processes that cause seasonal variations in surface‐ocean pCO_2_ solubility, wind speed, and mixed layer depth. Air‐sea CO_2_ flux seasonally alternates between CO_2_ outgassing (DIC loss from summer–fall) and uptake (DIC gain from winter–spring) in both hemispheres, with year‐round outgassing near the Equator (Figure 7b). Net biology produces a consistent loss of DIC at the Equator, with spring blooms resulting in seasonal DIC loss in both hemispheres.

#### 3.3.2 Seasonal Biome‐Scale Analysis

**Figure 8:**
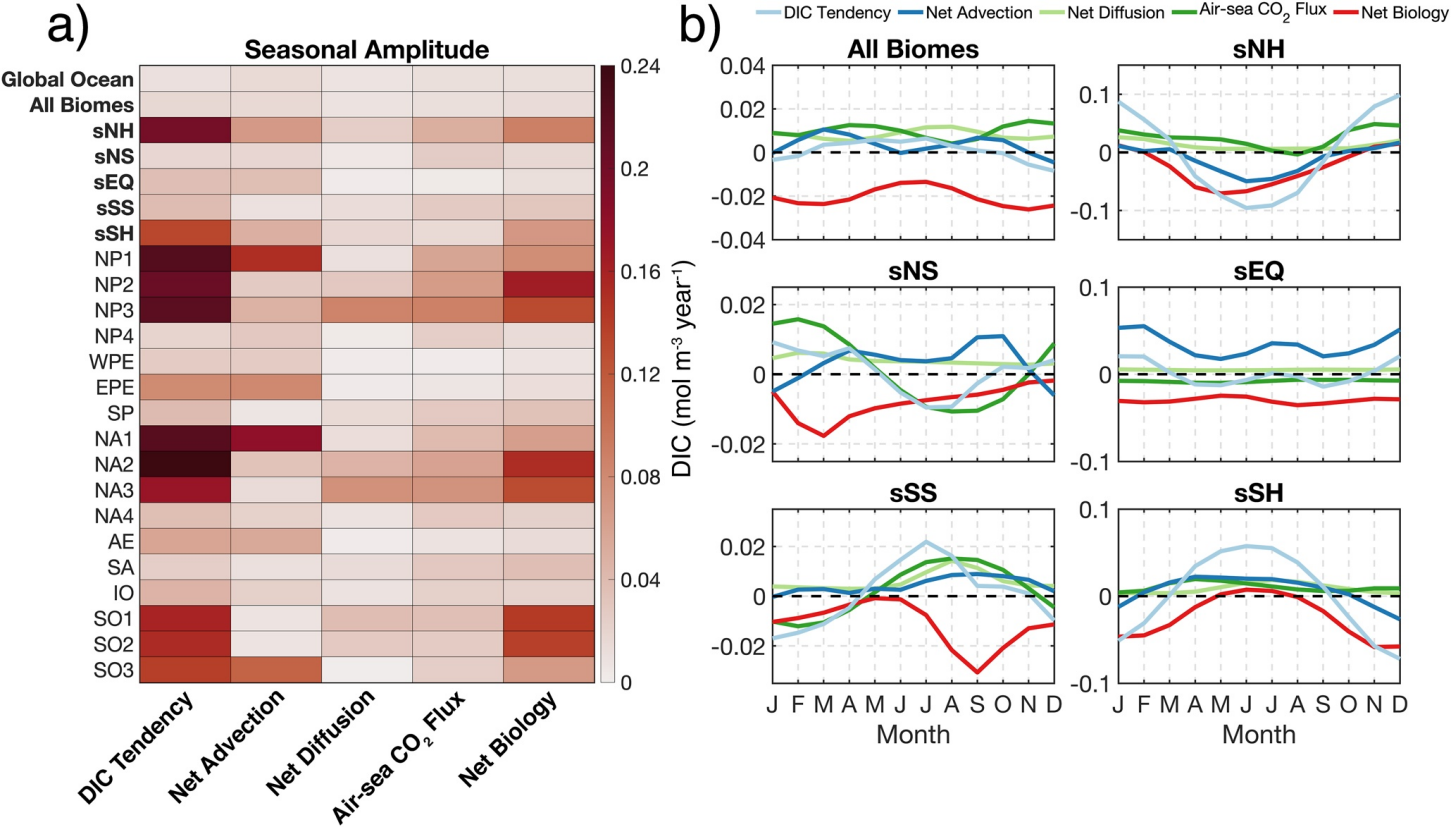
(a) Amplitude of climatological seasonal cycle for concentration budget terms in the upper 100 m for the global ocean and all biomes, superbiomes, and individual biomes. All values shown in (a) are listed in Table S1 in Supporting Information S1. (b) Seasonal climatology of concentration budget terms in the superbiomes; dashed black line shows the zero value. See Figure S3 in Supporting Information S1 for all individual biomes.

We now zoom into the biome scale to examine the amplitude of the climatological seasonal cycle for each budget term (Figure 8 and Table S1 in Supporting Information S1). Here the mass budget is normalized by volume to yield concentration, allowing for a comparison between biomes that is not biased by surface area. For all superbiomes, sNH has the largest DIC tendency amplitude (∼0.2 mol m^−3^ year^−1^), followed by sSH (Figure 8a). Seasonality in sNH results primarily from (in decreasing order) net biology, net advection, and air‐sea CO_2_ flux (Figure 8b). Examining the individual biomes that comprise sNH, we find that seasonality in high‐latitude NP1 and NA1 is largely modulated by net advection, with net biology playing a smaller role (see Figure S3 in Supporting Information S1). Here, summertime sea‐ice melt dilutes DIC‐rich waters, leading to divergence in net advection. Moving to the subpolar, seasonally stratified NP2 and NA2, the seasonal cycle is principally driven by strong net biology and air‐sea CO_2_ flux. Further south, in the subtropical seasonally stratified NP3 and NA3 biomes, which include the Gulf Stream and Kuroshio Current, net biology is weaker and net diffusion increases due to elevated vertical mixing.

In sSH, which is the Southern Ocean superbiome, DIC tendency is dominated by net biology (SO1 and SO2) and net advection along the periphery of Antarctica (SO3). Here, SO3 is strongly impacted by sea‐ice melt during austral summer (Figure S3 in Supporting Information S1). In equatorial sEQ, seasonality in DIC tendency is driven by time variability in net advection (WPE, EPE, and AE), which reaches local minima during the equinoxes; net biology and air‐sea CO_2_ flux have a weak seasonal cycle, with net diffusion having negligible seasonality (Figure 8b). For sSS, the seasonal cycle results from a combination of spring blooms (SP and SA), winter CO_2_ outgassing and summer uptake (SP, SA, and IO), and net advection in the Indian Ocean (IO). sNS, which contains permanently stratified NP4 and NA4, has the smallest DIC tendency amplitude of all superbiomes.

### 3.4 Budget Interannual Variability

#### 3.4.1 Global‐Ocean Upper‐100‐m Time Series

**Figure 9:**
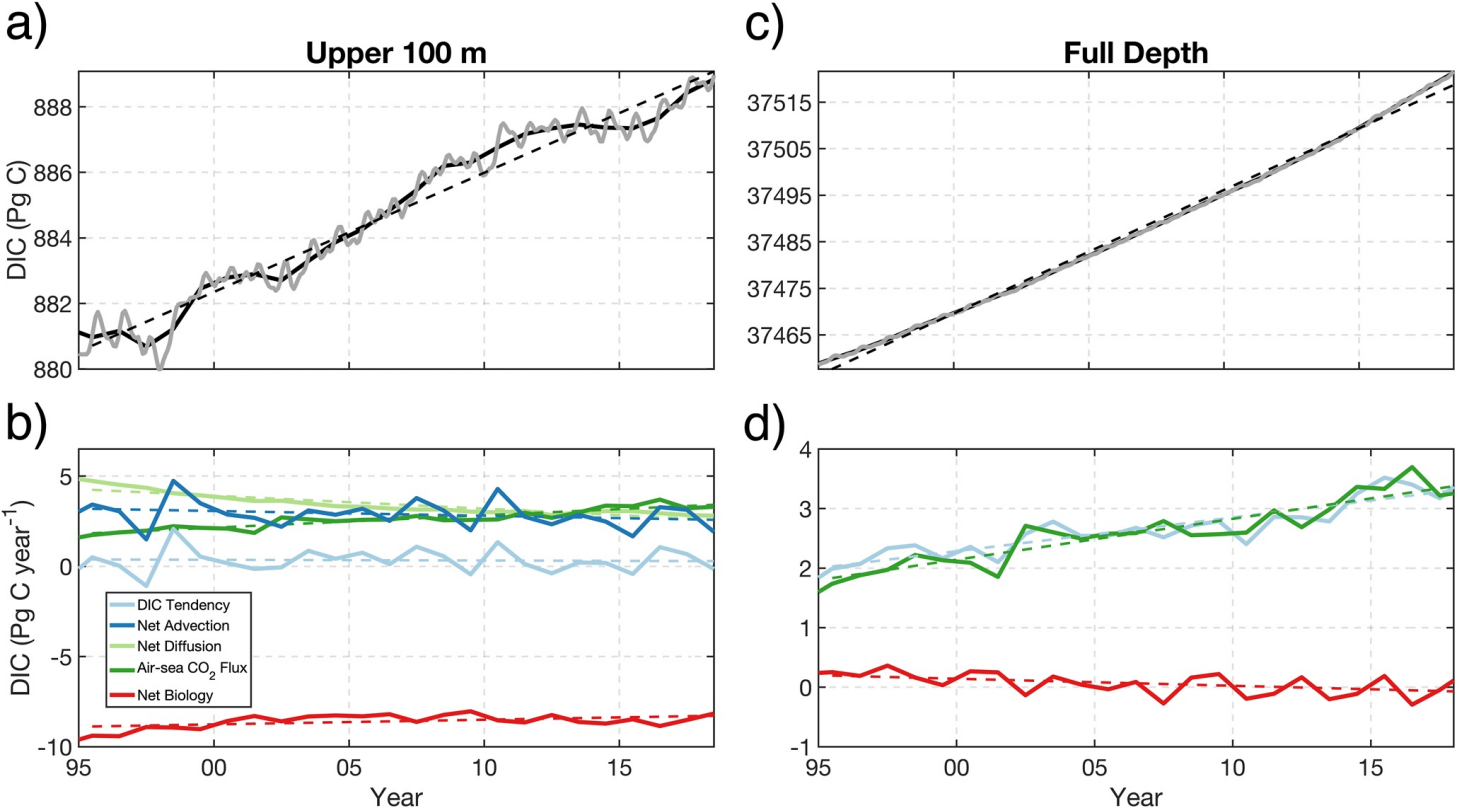
(a) Time series of dissolved inorganic carbon (DIC) content in the upper 100 m. Thick gray and black lines show monthly‐ and annual‐mean values, respectively. Thin dashed black line shows a linear fit to annual means. (b) Time series of annual‐mean mass budget terms for the upper 100 m. Thin dashed lines show linear fits to annual‐mean budget terms; dashed black line shows the zero value. DIC content and mass budget for the full‐depth ocean are shown in (c) and (d), respectively. Note that full‐depth net advection and net diffusion sum to zero when integrated globally and that PIC/POC removed at the sea floor is added to the full‐depth DIC budget.

Having shown climatological and seasonal results, we next examine global‐ocean interannual variability (IAV) in the upper 100 m and full‐depth DIC mass pool (Figure 9). DIC content in the upper 100 m exhibits strong IAV (Figure 9a), which is associated with the ENSO signal. ENSO events cause a sharp loss and subsequent gain in global‐ocean DIC mass during alternating El Niño and La Niña years, respectively (e.g., 1997–1998 and 2015–2016). Over the 24‐year model period, the 1997–1998 ENSO caused the largest global year‐to‐year change in upper‐ocean DIC mass, varying by 2.1 Pg C over two years. A least squares fit of annual‐mean DIC during 1995–2018 highlights the linear increasing trend in the upper ocean (Figure 9a, dashed black line). Over annual timescales, the upper‐100‐m DIC pool tracks the atmospheric CO_2_ growth rate, which to first‐order is linear over our model simulation. During 1995–2018, the upper‐100‐m DIC pool increased by 8.1 Pg C. Examination of the associated mass budget reveals that IAV in the upper‐100‐m DIC pool is primarily driven by net advection and is due to ENSO (Figure 9b). Air‐sea CO_2_ flux and net diffusion have opposing trends, as the secular increase in ocean CO_2_ uptake reduces upper‐ocean carbon gradients and weakens vertical mixing of DIC. Over the 24‐year model period, biological DIC loss in the upper 100m weakened by roughly 6.7%.

#### 3.4.2 Global‐Ocean Full‐Depth Time Series

For the full‐depth DIC pool (Figure 9c), IAV from ENSO events has a negligible impact on the large ocean reservoir, with a small seasonal cycle driving subannual variability (gray line). Because of increasing atmospheric CO_2_ concentration, the global‐ocean DIC pool cumulatively sequesters increasing atmospheric CO_2_, resulting in a slightly nonlinear increase of DIC mass. Over the model period, the global‐ocean DIC pool increased from 37,455 to 37,519 Pg C (or Gt C), a difference of roughly 64 Pg C (∼2.7 Pg C year^−1^). In the corresponding full‐depth budget (Figure 9d), the secular increase in DIC tendency, which increases by roughly 64% over the model period, is driven primarily by the linear increase in air‐sea CO_2_ flux. Full‐depth net biology provides a small contribution that is generally centered around zero (time mean of 0.1 Pg C year^−1^).

We note that net advection and diffusion fully compensate when integrated across the full‐depth global ocean, that is, circulation only redistributes DIC and does not result in a net gain or loss. Also note that PIC/POC removed at the model seafloor is added to the full‐depth DIC budget in Figures 9c and 9d. This is because in the real ocean, particulate carbon is expected to either be remineralized and dissolved before being buried or to be replenished by riverine fluxes, processes that are not currently represented in ECCO‐Darwin.

#### 3.4.3 Biome‐Scale Time Series

**Figure 10:**
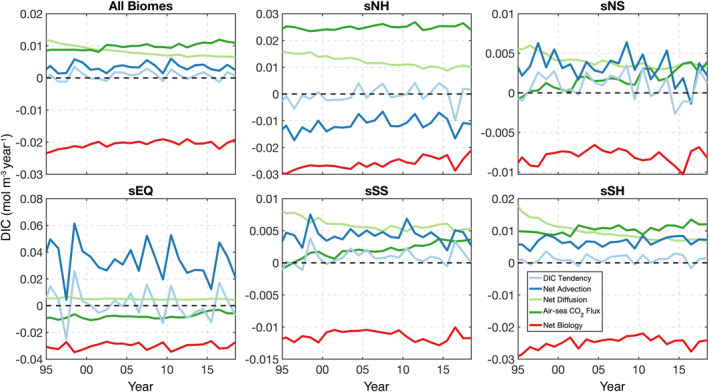
Annual‐mean time series of concentration budget terms in the upper 100 m for all superbiomes; dashed black line shows the zero value. See Figure S4 in Supporting Information S1 for all individual biomes.

Having shown the global‐ocean results, we next focus again on the biome scale to examine regional IAV. Annual‐mean time series of concentration budget terms (Figure 10), taken in the upper 100 m of all biomes, demonstrates that DIC tendency (light blue line) closely tracks IAV in net advection (dark blue line). Over the 24‐year model period, DIC tendency is generally positive (DIC gain) when averaged across all superbiomes, only becoming slightly negative (DIC loss) during major ENSO events. sNH exhibits negative net advection during the entire model period; the magnitude of this DIC loss results from consistent advective divergence in NA2 and NA3, with associated IAV driven by ice‐influenced NP1 and NA1 (Figure S4 in Supporting Information S1). In sNS, DIC tendency is also dominated by sharp year‐to‐year variability in net advection, with IAV in air‐sea CO_2_ flux and net biology playing a secondary role.

Equatorial sEQ shows the clear influence of ENSO events and other long‐term fluctuations. Here net advection undergoes a sharp reduction during El Niño years due to arrested equatorial upwelling in WPE and EPE (Figure S4 in Supporting Information S1). The reduction in (a) net advection of DIC‐rich waters to the mixed layer and (b) nutrients to the euphotic zone during El Niño (not shown) causes a corresponding decrease in CO_2_ outgassing and biological uptake, respectively. We note that the very strong decrease in net advection causes only a minor reduction in net outgassing, due to compensation from thermal effects (i.e., warmer waters cause more outgassing of natural CO_2_) and anthropogenic CO_2_ uptake (reduced upwelling results in less anthropogenic CO_2_ uptake). In WPE, the 2015 El Niño reduced CO_2_ outgassing enough that short‐term uptake was achieved. Interestingly, net diffusion in sEQ does not exhibit the strong secular decline seen in the other superbiomes; this is because CO_2_ outgassing helps maintain the vertical structure of the carbocline, holding net diffusion steady. We find that DIC tendency in sSS is also impacted by major ENSO events (e.g., 1997–1998 and 2015–2016), highlighting the reduced upwelling and poleward advection occurring in both sEQ and sSS.

In Southern Ocean sSH, IAV is driven by a combination of net advection, air‐sea CO_2_ flux, and net biology. Here IAV predominately occurs in SO2 and SO3 (Figure S4 in Supporting Information S1), which are located closer to the Antarctic continent. The sum of terms with DIC gain generally exceeds the loss from net biology, except during 1996, 2002, 2009, and 2016; years when net advection in the ice‐dominated SO3 decreased sharply (Figure S4 in Supporting Information S1), leading to a negative DIC tendency.

#### 3.4.4 Biome‐Scale Magnitudes and Trends

**Figure 11:**
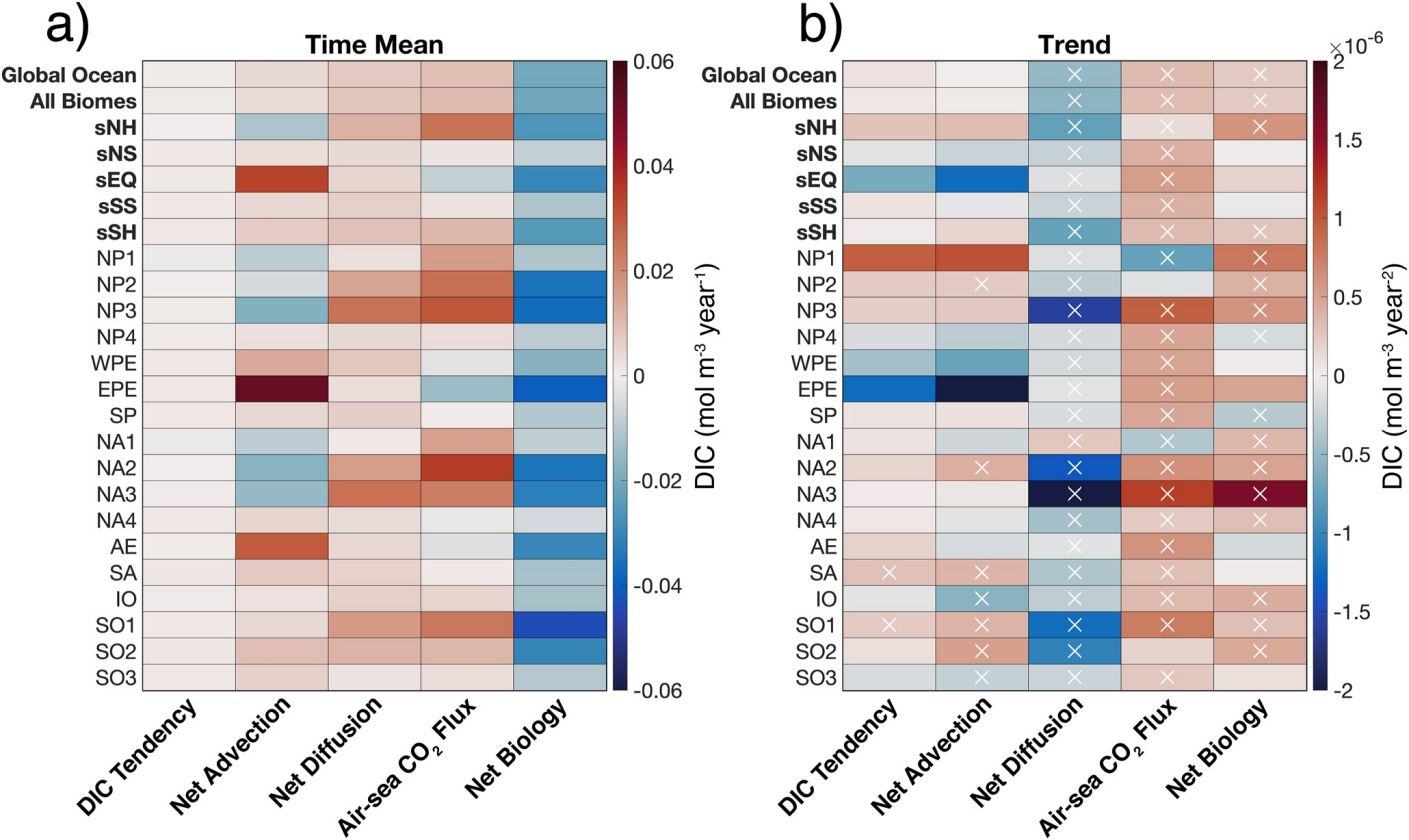
(a) Time‐mean of concentration budget terms in the upper 100 m of the global ocean and all biomes, superbiomes, and individual biomes during 1995–2018. (b) Corresponding linear trends computed from a least squares regression; white crosses show trends that are statistically significant at the 95% confidence level. All values shown in (a) are listed in Table S2 in Supporting Information S1.

Finally, we evaluate biome‐scale budget magnitudes and trends, computed from annual‐mean concentration budget time series in the upper 100 m (Figure 11 and Table S2 in Supporting Information S1). The small time‐mean DIC tendency values, which are roughly an order of magnitude smaller than the other terms, again highlight the substantial compensation between bio‐chemical‐physical processes (Figure 11a).

For all superbiomes, equatorial sEQ has the strongest time‐mean net advection, resulting from vigorous upwelling of DIC‐rich waters along the Equator in EPE and AE. Maximum net advection (DIC gain of ∼0.05 mol m^−3^ year^−1^) is obtained in EPE, which is located in the eastern Equatorial Pacific Ocean. In contrast, Northern Hemisphere subpolar and subtropical seasonally stratified biomes (NP2, NP3, NA2, and NA3) have negative time‐mean net advection, primarily driven by the advective divergence of DIC in boundary current systems and their poleward‐flowing extensions. Ice‐influenced biomes NP1 and NA1 also exhibit negative net advection, caused by the dilution of DIC‐rich waters from sea‐ice melt during summer. All superbiomes have positive time‐mean net diffusion, with sNH having the largest diffusive gain of DIC (∼0.01 mol m^−3^ year^−1^), due to elevated vertical mixing in the subpolar and subtropical seasonally stratified biomes (NP2, NP3, NA2, and NA3). Positive air‐sea CO_2_ flux (uptake) is found in all superbiomes except for equatorial sEQ, which has a negative time‐mean flux (outgassing). A maximum superbiome air‐sea CO_2_ flux of ∼0.02 mol m^−3^ year^−1^ occurs in sNH, driven by strong CO_2_ uptake in NP3 and NA2. For all individual biomes, maximum and minimum air‐sea CO_2_ fluxes occur in North Atlantic NA2 and equatorial EPE, respectively. Time‐mean net biology is negative (DIC loss) in all superbiomes, with the largest magnitudes (in decreasing order) occurring in sEQ, sNH, and sSH. For all individual biomes, the strongest biological uptake occurs in Southern Ocean SO1 (∼0.04 mol m^−3^ year^−1^).

The majority of statistically significant trends (95% confidence interval) occur in net diffusion, air‐sea CO_2_ flux, and net biology terms (Figure 11b, white crosses show p‐values ≤ 0.05). For all biomes, significant trends in DIC tendency occur only in South Atlantic SA and Southern Ocean SO1, with both biomes having a positive trend. Significant trends in net advection are obtained in 7/17 biomes, with Indian Ocean IO and Southern Ocean SO3 having the only negative trends. All superbiomes and biomes have statistically significant trends in net diffusion, with negative trends in all biomes except for the ice‐influenced NA1. The majority of biomes (15/17) demonstrate significant trends in air‐sea CO_2_ flux, with Northern Hemisphere ice‐influenced NP1 and NA1 having the only significant negative trends. Significant trends in net biology occur in sNH and sSH, and in roughly 70% of biomes (12/17). Only NP4 and SP have significant negative trends in net biology (i.e., increasing biological DIC loss).

## 4 Discussion

### 4.1 Overview

Data‐based reconstructions provide increasingly accurate estimates of the ocean sink for atmospheric CO_2_. These reconstructions, however, do not fully explain the mechanisms that drive space‐time variability in the sink. Alternatively, hindcast OBMs provide a tool for exploring the individual processes that control ocean carbon variability; however, most of these models are not constrained by observations. Using the ECCO‐Darwin global‐ocean biogeochemistry state estimate, we have combined the above two methods and produced a multi‐decadal (1995–2018), data‐constrained DIC budget that fully attributes ocean sink variability to three‐dimensional physical, chemical, and biological drivers.

**Figure 12:**
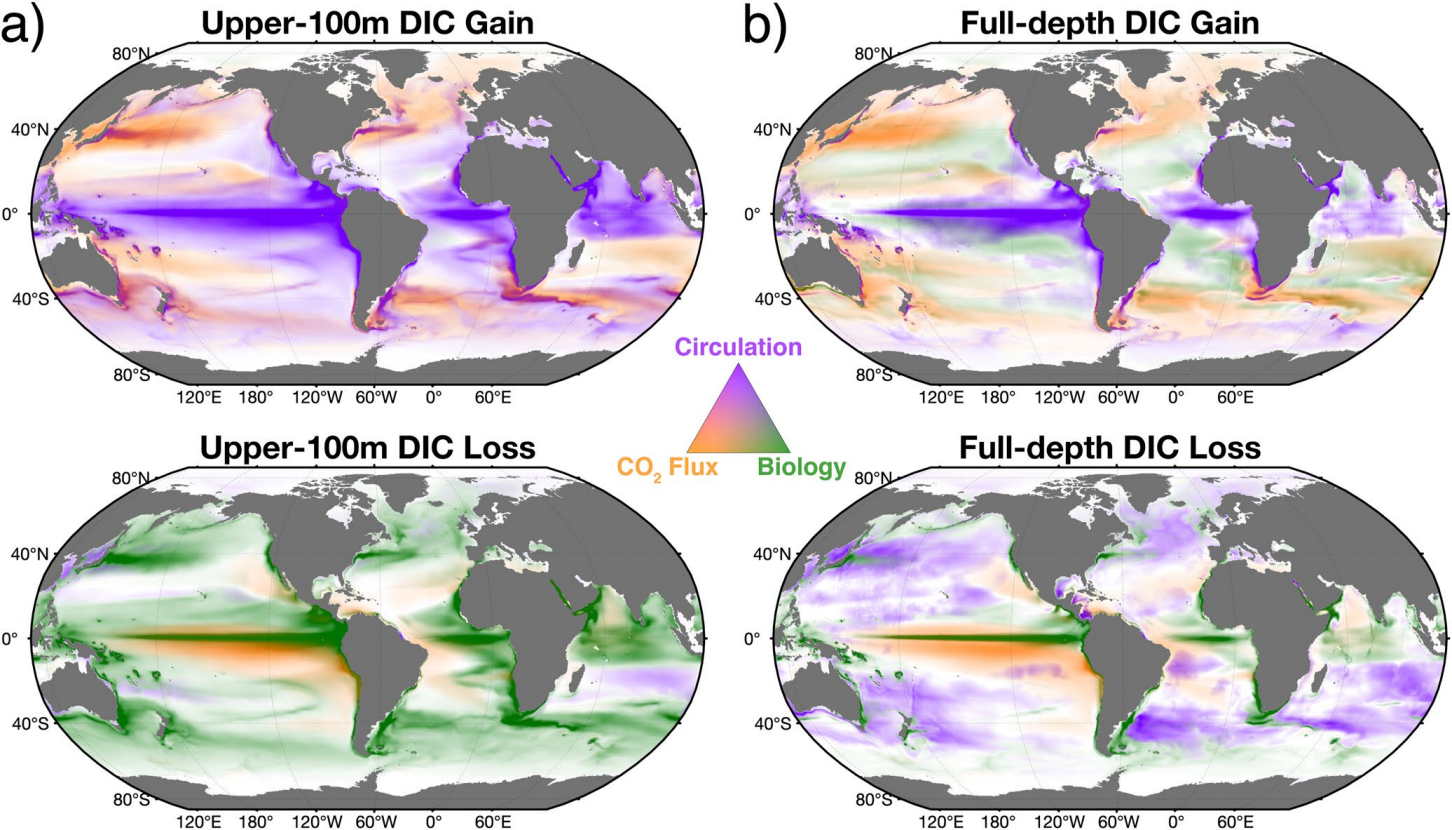
Climatological ternary maps for (a) the upper 100 m and (b) full‐depth ocean. Ternary maps show blended DIC gain (top row) and loss (bottom row) from ocean circulation (purple colors), air‐sea CO_2_ flux (orange colors), and net biology (green colors). Circulation represents the sum of net advection and net diffusion. Ternary maps are computed from vertically integrated mass budget terms; color saturation shows the relative magnitude of the term.

The fully closed decomposition of DIC budget terms is a powerful tool for explaining observed DIC variability. In the upper ocean (top 100 m in this study), advective and diffusive processes (i.e., circulation) provide the largest gain of DIC, with net biology resulting in the largest loss. When considering the volume‐integrated global ocean, air‐sea CO_2_ flux provides the largest DIC gain (time mean and standard deviation of ∼2.6 ± 0.5 Pg C year^−1^, respectively). Globally, interannual variability is dominated by vertical advection in equatorial regions, with ENSO causing the largest year‐to‐year change in upper‐ocean DIC mass. In terms of the altered modern vertical DIC gradient, we note that a natural DIC flux, which has similar magnitude to the loss by net biology, is brought to the surface by advective and diffusive processes (i.e., ocean circulation). However, there is also a residual downward anthropogenic DIC flux from air‐sea CO_2_ exchange. Assuming the imbalance between circulation and net biology roughly reflects the net downward anthropogenic DIC flux (2.3 Pg C year^−1^), there would be a physical anthropogenic carbon removal of 55 Pg C to depths greater than 100 m over the 24‐year model period. This accounts for 85% of the full‐depth DIC mass increase over the same period, with ∼8 Pg C remaining in the upper 100 m. We next summarize the time‐mean, blended patterns of DIC gains and losses across the global ocean (Figure 12).

1. **Circulation**

In the upper 100‐m, ocean circulation (Figure 12a, purple colors), which is the sum of net advection and net diffusion, is the primary gain of DIC in equatorial regions, western boundary currents, and along eastern boundary current upwelling zones (e.g., California, Peru, Benguela, and Canary Current systems). While we note that diffusion represents unresolved turbulent processes and the split between advection and diffusion depends on model configuration and the choice of the 100 m vertical integration depth, we hypothesize that the combined circulation component is robust. For circulation in western boundary currents, diffusion exceeds advection, while in the equatorial and upwelling regions advection dominates circulation (Figures 5b and 5c).

When examining the full‐depth ocean (Figure 12b), gains of DIC from intense swaths of circulation are reduced in size, due to substantial compensation of advective and diffusive processes throughout the water column. In the upper 100 m, circulation results in a weak loss of DIC only in few select regions (e.g., subpolar North Atlantic and Indian Oceans). For the full‐depth ocean, DIC loss from circulation is substantial outside of equatorial regions, highlighting the large‐scale extratropical divergence required to balance equatorial convergence of DIC‐rich waters.

2. **Air‐sea** **CO**_**2**_ **Flux**

Ocean‐atmosphere exchange of natural and anthropogenic CO_2_  (Figure 12, orange colors), which is the same in the upper 100 m and full‐depth ocean, results in a gain of DIC in western boundary currents, subpolar regions, select subtropical regions (e.g., Pacific and Indian Oceans), and along the Subantarctic Front of the Antarctic Circumpolar Current (ACC). For subtropical western boundary currents, warm waters are transported poleward, which leads to dramatic cooling and a subsequent increase in CO_2_ solubility. Meanwhile, subpolar boundary currents bring cold waters equatorward, which then warm and hence outgas CO_2_. Additionally, subtropical western boundary currents are regions exhibiting strong biological loss of DIC, which allows for larger air‐sea pCO_2_ gradients and hence increased CO_2_ uptake. Air‐sea CO_2_ flux drives a loss of DIC in equatorial regions, where upwelling of DIC‐rich waters from circulation results in intense CO_2_ outgassing, and in broad swaths offshore of several eastern boundary currents (e.g., California and Peru Current systems).

3. **Biological Processes**

In the upper 100 m, biological processes (Figure 12a, green colors) result in a loss of DIC due to fixation of carbon in the euphotic zone and export of particulate carbon to depth. Hotspots of DIC loss from upper‐ocean biology directly overlap with circulation‐driven DIC gain as upwelling and vertical mixing of carbon also delivers vital nutrients to the euphotic zone, fueling biological productivity. The upwelling that causes strong productivity also results in horizontal divergence away from highly productive regions.

In the full‐depth water column, subtropical regions are locations of DIC gain (Figure 12b). This is primarily due to lateral convergence of dissolved organic carbon (DOC), which is then remineralized away from regions where it was produced, that is, regions of high biological productivity. While particulate organic carbon (POC) sinks and is remineralized below the export region, DOC remains in suspension and is advected laterally to the center of the subtropical gyres. Compared to the upper 100 m, full‐depth biological loss is reduced in both intensity and surface area due to DOC being transported and remineralized away from regions of high productivity.

### 4.2 Global‐Ocean DIC Mass

The simulated global‐ocean DIC mass from ECCO‐Darwin (time mean of 37,484 Pg C) agrees well with previous broad‐scale estimates of the inorganic carbon pool (37,400 Pg C in Falkowski et al., 2000) and recent data‐based reconstructions (37,214 ± 195 Pg C in Keppler et al., 2020). Additionally, our results suggest a full‐depth DIC gain of ∼64 Pg C between 1995 and 2018, with air‐sea CO_2_ flux contributing a total of 63 Pg C and the biological pump contributing 1.4 Pg C (Figure 9d). In the upper 100‐m, we estimate a smaller ocean sink increase of 8.1 Pg C over the model period, accounting for roughly 13% of the full‐depth increase. In the same depth range, net biology result in a net community production (NCP; Sarmiento & Gruber, 2006) of 8.6 Pg C year^−1^, which is in line with previous observation‐based (Lee, 2001; Li & Cassar, 2016) and data‐assimilative model (9 Pg C year^−1^; DeVries & Weber, 2017) estimates, lending confidence to our estimates of global‐ocean productivity. Furthermore, our ocean CO_2_ sink trajectory is generally consistent with reconstructions derived from observations (Gruber et al., 2019; Khatiwala et al., 2009). The recent estimate from Gruber et al. (2019) suggests a net ocean CO_2_ uptake (natural and anthropogenic) of 29 ± 5 Pg C between 1994 and 2007. When computed over the 1995–2007 period, our simulation results in a global‐ocean DIC mass increase of roughly 31 Pg C (Figure 9d), consistent with the Gruber et al. (2019) estimate.

These results highlight the utility of using a global‐ocean biogeochemistry state estimate, such as ECCO‐Darwin, to: (a) complement data‐based reconstructions and quantify the individual mechanisms that modulate ocean sink variability (McKinley et al., 2017); and (b) provide critical constraints on the long‐term impact of the terrestrial biosphere on atmospheric CO_2_ concentrations (Friedlingstein et al., 2019). Additionally, our results from the 1995–2007 period suggest that 53% of the atmospheric CO_2_ uptake between 1995 and 2018 occurred after 2007, with the later 11 years having a time‐mean CO_2_ exchange rate of roughly 3 Pg C year^−1^. Finally, we note that the Green's Functions approach described in Carroll et al. (2020), combined with sensitivity experiments that separate the global‐ocean DIC inventory into natural and anthropogenic components (DeVries, 2014; Sabine et al., 2004), could be used to gain a more complete understanding of natural and forced variability in the ocean sink (McKinley et al., 2020).

### 4.3 Interannual Variability and ENSO

Globally, we find that the long‐term trend and IAV in upper‐ocean DIC is driven principally by (a) the growth rate of atmospheric CO_2_ and (b) major ENSO events, respectively (Figure 9a); results that are supported by previous model‐based studies (McKinley et al., 2004, 2020; Resplandy et al., 2015). We also note that our climatological DIC tendency of 0.3 Pg C year^−1^ generally matches observation‐based estimates of ∼1 *μ*mol kg^−1^ year^−1^ (Gregor & Gruber, 2021). In the Pacific Ocean between 15°S and 8°N, vigorous outgassing of CO_2_ drives a consistent loss of DIC during ENSO‐neutral years (Figure 6 and Figure S4 in Supporting Information S1). This CO_2_ outgassing is stronger on the eastern side of the basin (Figure 5d), where ocean‐atmosphere pCO_2_ gradients are large (Carroll et al., 2020; Landschützer et al., 2016; Sutton et al., 2014). However, during major El Niño events, equatorial upwelling of DIC‐rich waters is substantially arrested and net advection decreases, causing an associated decline in CO_2_ outgassing and biological processes (Figure 10, see sEQ). The changes in carbon exchange were large enough during the 2015–2016 El Niño event that they were observed by OCO‐2 (Chatterjee et al., 2017; Patra et al., 2017). During La Niña, equatorial upwelling is reinvigorated, with CO_2_ outgassing and biological productivity rebounding sharply.

**Figure 13:**
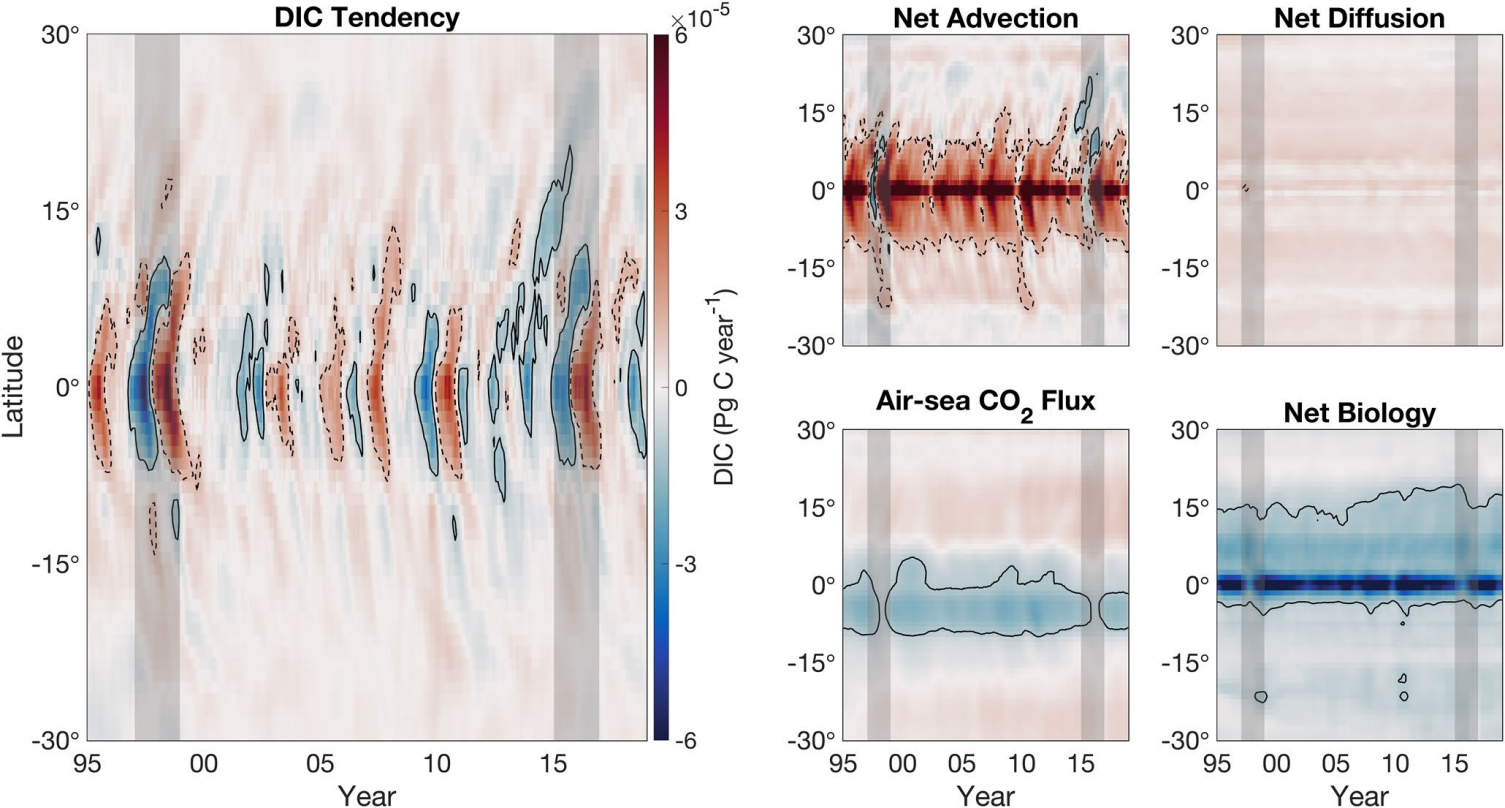
Hovmöller diagram of mass budget terms in Pacific Ocean biomes (NP2, NP3, NP4, WPE, EPE, and SP), zonally averaged in the upper 100 m from 30°S to 30°N. Monthly mean budget terms are smoothed with a 12‐month, centered running mean. Vertical gray lines show the 1997–1998 and 2015–2016 ENSO events. Solid black contours represent DIC loss and dashed black lines show DIC gain; a contour magnitude of 10^−6^ Pg C year^−1^ is used for both.

Examination of the Pacific Ocean DIC budget in Hovmöller space (Figure 13), demonstrates the wide range of bio‐physical ENSO diversity (Capotondi et al., 2015; Gierach et al., 2012) captured by our simulation. We find that during the major 1997–1998 and 2015–2016 El Niño events (vertical gray lines), net advection along the Equator changes sign and transitions from a DIC gain to a weak loss. Time‐depth Hovmöller diagrams, taken in WPE and EPE, highlight the asymmetrical west‐to‐east response of the equatorial carbocline and budget terms to diverse ENSO events (Figure S5 in Supporting Information S1).

In west equatorial WPE, the carbocline is deeper and is capped by low‐DIC waters associated with the warm pool (Feely et al., 2002). During the large 1997–1998 and 2015–2016 ENSO events, WPE exhibits increased upwelling of DIC‐rich waters and biological production; this reduces outgassing to near zero (Figure S5a in Supporting Information S1). An opposing set of changes occurs in east equatorial EPE during ENSO, with El Niño conditions resulting in a sharp loss of DIC in the upper 100 m, which corresponds to arrested equatorial upwelling, reduced outgassing of CO_2_, and a decrease in biological production and deep remineralization (Figure S5b in Supporting Information S1). Additionally, our results show that advective ENSO signals along the Equator are propagated poleward by Ekman transport and subsequent flow into the subtropical gyres (Figure 10, see net advection in sEQ and sSS).

### 4.4 Observed Trend or Model Drift?

There are two standard ways of initializing an ocean biogeochemistry model. The first is to start the simulation close to observations, for example, a climatology, which can result in a large model drift over time. The second is to start the simulation after a long spin‐up, which can result in a large model bias relative to observations. The third approach, and the one that we adopt here for both the physics and biogeochemistry, is to use data assimilation to obtain a solution that simultaneously minimizes model bias and drift, according to an objective cost function. Rather than spin‐up the model for thousands of years to a preindustrial steady‐state before applying an anthropogenic CO_2_ perturbation, we aim to obtain 1995 initial conditions that have small bias compared to observations at depth (see Figure 4c and Figure S1 in Supporting Information S1) while at the same time minimizing the upper‐ocean initialization shock. Because of the low‐dimensionality of the biogeochemical estimation (i.e., small number of control parameters), we further reduce initialization shock by discarding the first 3 years (1992–1994) of the simulation (Figure S6 in Supporting Information S1).

The dashed lines in Figure 9 show simulated trends during 1995–2018. A key question is whether these trends are real or result from residual model drift. Although we have used data assimilation to minimize model bias and drift, we acknowledge that the drift of some of the budget terms, for example, the net diffusion or net biology terms in Figures 9b and 10, may be a residual model spin‐up issue as opposed to a real signal.

The net diffusion term for the upper 100 m of the global ocean (Figure 9b, light green line) has a 1995–2018 trend of −72 TgC year^−2^. This trend is primarily caused by the increasing anthropogenic perturbation of atmospheric CO_2_, which tends to decrease the vertical gradient of total DIC in the upper ocean. We have verified this via a sensitivity experiment that reduced apCO_2_, maintaining the observed 1995 seasonal cycle for the complete 1995–2018 period. In this sensitivity experiment with lower apCO_2_, the diffusion term contribution to upper‐100‐m DIC mass is higher, that is, it decreases more slowly during the 1995–2018 integration period, as would be the case when a sharper vertical DIC gradient is maintained. Note that the decreasing net diffusion trend is accompanied by increasing CO_2_ flux trend (Figures 9b and 10, dark green line). Also note that the Equatorial region, where there is little anthropogenic CO_2_ uptake, has no trend in the diffusion term of the upper 100 m (see sEQ in Figure 10).

The net biology term for the upper 100 m of the global ocean (Figure 9b, red line) has a 1995–2018 trend of 26 TgC year^−2^. Contrary to the net diffusion term, there is no immediate known cause for such a trend. A 1995–2018 model sensitivity experiment where the nutrients were reinitialized from end‐of‐2018 conditions (Figure S7 in Supporting Information S1) indicates that model drift may, in part, be the cause for this trend. However, the biology trend is small relative to interannual variability and it is only noticeable for globally integrated quantities. In all cases, there are insufficient observations to verify globally integrated, model‐predicted trends. These model predictions will need to be confirmed or invalidated by future observational or modeling studies.

We also stress that the choice of vertical integration depth for the DIC mass budget affects compensation and hence the relative importance of budget terms. The Southern Ocean DIC budget study of Rosso et al. (2017) used a depth of 650 m, which was chosen to span the deepest modeled mixed layer. We find that using a shallower integration depth of 100 m results in increased net diffusion (due to atmospheric CO_2_ uptake), a term which was shown to have a small impact in Rosso et al. (2017). Globally, we find that depth‐integrated net advection exceeds net diffusion at depths greater than 127 m (not shown). The points raised above should be considered when interpreting our upper‐ocean budget results.

### 4.5 Model Caveats and Potential Improvements

In addition to being a powerful tool for explaining observed DIC variability, the decomposition of DIC budget terms can also be used to identify model deficiencies and their causes. A partial list of known model deficiencies are enumerated below as a focus for future work. Our treatment of freshwater runoff along the coastal zone, and from the Greenland and Antarctic Ice Sheets, is coarsely represented and does not capture IAV (Fekete et al., 2002). Furthermore, we do not include biogeochemical runoff (i.e., DIC and nutrients, Mayorga et al., 2010), which may be critical for accurate simulation of Arctic Ocean biogeochemistry (Le Fouest et al., 2013, 2015). Future efforts should incorporate time‐varying, point‐source global freshwater (Feng et al., 2021) and biogeochemical runoff (Resplandy et al., 2018) to improve the simulated ocean sink in coastal (Chen et al., 2013) and high‐latitude regions (Hopwood et al., 2018, 2020; Wadham et al., 2019). Finally, ECCO‐Darwin does not contain an explicit representation of bottom sediment dynamics. To balance the input of riverine carbon and to help address large uncertainties in carbon burial rates (Dunne et al., 2007), future efforts should include a realistic representation of burial and sediment‐water fluxes, that is, add a bottom sediment model (Sulpis et al., 2021) and optimize dissolution, remineralization, and particle‐sinking rates.

The ECCO‐Darwin biogeochemical optimization also relies on a low‐dimensional (i.e., small number of control variables) Green's Functions approach to minimize model‐data misfit (Carroll et al., 2020; Menemenlis et al., 2005). The incorporation of an adjoint‐based optimization method, as done in Verdy and Mazloff (2017) and Rosso et al. (2017) for the Southern Ocean, would allow for a larger number of control variables and joint physical‐biogeochemical optimization, which could further improve the fit of simulated DIC to observations. Future work will incorporate the Darwin spectral light and radiative transfer package (Dutkiewicz et al., 2015, 2018, 2019), which will allow for the assimilation of satellite ocean color data. Combined with in‐situ particle flux observations (Estapa et al., 2019; Siegel et al., 2016), this would: (a) provide critical constraints on simulated phytoplankton dynamics, export, and remineralization rates; and (b) improve the model representation of climate‐driven variability in biological export, for example, decline in primary productivity due to increased stratification (Lozier et al., 2011).

Finally, we stress that further model‐data uncertainty analysis is required to confirm the multi‐decadal trends evident in our budget analysis (Figure 10b). We note, however, that our use of data‐constrained circulation estimates from ECCO lends credibility to the simulated space‐time variability in advective and diffusive budget terms. Furthermore, ECCO‐Darwin surface‐ocean pCO_2_ and air‐sea CO_2_ flux has been extensively evaluated against leading data‐based reconstructions (Landschützer et al., 2016; Rödenbeck et al., 2015) and shows broad‐scale consistency across most biomes, particularly in the subtropical and equatorial regions (Carroll et al., 2020). As mentioned previously, further efforts and comparison with observations are needed to confirm that trends in our simulated biology are driven by natural variability. Additionally, we acknowledge that the nominal 1/3° horizontal grid spacing of ECCO‐Darwin does not permit explicit representation of mesoscale eddies and submesoscale processes. As it becomes computationally practical, we expect that increased model resolution will allow for representation of fine‐scale, bio‐physical interactions (Mahadevan, 2016; Whitt et al., 2019).

## 5 Concluding Remarks

We have produced a data‐constrained, global‐ocean DIC budget that permits attribution of space‐time variability in the ocean sink to three‐dimensional ocean circulation, ocean‐atmosphere CO_2_ exchange, and biological processes. The data‐constrained DIC budget presented here represents a significant advance and clearly demonstrates the intricate spatial diversity and compensation in ocean sink mechanisms. These results provide much‐needed context for sparse in‐situ biogeochemical observations and can be used to inform data‐based reconstructions of the global‐ocean DIC pool and ocean sink trajectory. The ECCO‐Darwin solution, the budget terms, and all software used to generate the simulation and diagnostics have been made available to the science community and are expected to enable further global and regional biogeochemical studies. As (a) the fidelity of ECCO physical‐ocean circulation estimates continue to improve and extend in time and (b) upper‐ocean biogeochemical observations become more numerous (e.g., from BGC‐Argo floats), we anticipate that our data‐constrained DIC budget will be an ever more useful tool for ocean carbon sink, ecosystem, and climate‐related studies. Ultimately, this work supports the future development of a fully coupled ocean‐atmosphere carbon state estimate.

## Erratum

The sentence on page 17 that currently begins “Air‐sea CO2 flux drives a loss of DIC in equatorial regions…” used the word “gain” instead of “loss” due to a typographical error. The typographical error, which has now been corrected, did not affect the results or conclusions. This may be considered the official version of record.

## Supporting information

Supporting Information S1

## Data Availability

ECCO‐Darwin model output and DIC budget diagnostics are available at the ECCO Data Portal: http://data.nas.nasa.gov/ecco/. Model code and platform‐independent instructions for running ECCO‐Darwin simulations are available at: https://zenodo.org/record/6091603.

## References

[gbc21250-bib-0001] Andrews, A. E. , Kofler, J. D. , Trudeau, M. E. , Williams, J. C. , Neff, D. H. , Masarie, K. A. , et al. (2014). CO_2_, CO, and CH_4_ measurements from tall towers in the NOAA Earth system research laboratory’s global greenhouse gas reference network: Instrumentation, uncertainty analysis, and recommendations for future high‐accuracy greenhouse gas monitoring efforts. Atmospheric Measurement Techniques, 7(2), 647–687. 10.5194/amt-7-647-2014

[gbc21250-bib-0002] Archer, D. , Eby, M. , Brovkin, V. , Ridgwell, A. , Cao, L. , Mikolajewicz, U. , et al. (2009). Atmospheric lifetime of fossil fuel carbon dioxide. Annual Review of Earth and Planetary Sciences, 37(1), 117–134. 10.1146/annurev.earth.031208.100206

[gbc21250-bib-0003] Aumont, O. , Ethé, C. , Tagliabue, A. , Bopp, L. , & Gehlen, M. (2015). PISCES‐v2: An Ocean biogeochemical model for carbon and ecosystem studies. Geoscientific Model Development, 8(8), 2465–2513. 10.5194/gmd-8-2465-2015

[gbc21250-bib-0004] Bakker, D. C. E. , Pfeil, B. , Landa, C. S. , Metzl, N. , O’Brien, K. M. , Olsen, A. , & Xu, S. (2016). A multi‐decade record of high‐quality *f*CO_2_ data in version 3 of the Surface Ocean CO_2_ Atlas (SOCAT). Earth System Science Data, 8(2), 383–413.

[gbc21250-bib-0005] Bloom, A. A. , Bowman, K. W. , Liu, J. , Konings, A. G. , Worden, J. R. , Parazoo, N. C. , et al. (2020). Lagged effects regulate the inter‐annual variability of the tropical carbon balance. Biogeosciences, 17(24), 6393–6422. 10.5194/bg-17-6393-2020

[gbc21250-bib-0006] Bowman, K. W. , Liu, J. , Bloom, A. A. , Parazoo, N. C. , Lee, M. , Jiang, Z. , et al. (2017). Global and Brazilian carbon response to El Niño Modoki 2011‐2010. Earth and Space Science, 4(10), 637–660. 10.1002/2016ea000204

[gbc21250-bib-0007] Boyd, P. W. , Claustre, H. , Levy, M. , Siegel, D. A. , & Weber, T. (2019). Multi‐faceted particle pumps drive carbon sequestration in the ocean. Nature, 568(7752), 327–335. 10.1038/s41586-019-1098-2 30996317

[gbc21250-bib-0008] Brix, H. , Menemenlis, D. , Hill, C. N. , Dutkiewicz, S. , Jahn, O. , Wang, D. , et al. (2015). Using Green’s Functions to initialize and adjust a global, eddying ocean biogeochemistry general circulation model. Ocean Modelling, 95, 1–14. 10.1016/j.ocemod.2015.07.008

[gbc21250-bib-0009] Campin, J.‐M. , Marshall, J. , & Ferreira, D. (2008). Sea ice–ocean coupling using a rescaled vertical coordinate z*. Ocean Modelling, 24(1), 1–14. 10.1016/j.ocemod.2008.05.005

[gbc21250-bib-0010] Capotondi, A. , Wittenberg, A. T. , Newman, M. , Di Lorenzo, E. , Yu, J. Y. , Braconnot, P. , et al. (2015). Understanding ENSO diversity. Bulletin of the American Meteorological Society, 96(6), 921–938. 10.1175/bams-d-13-00117.1

[gbc21250-bib-0011] Carroll, D. , Menemenlis, D. , Adkins, J. F. , Bowman, K. W. , Brix, H. , Dutkiewicz, S. , et al. (2020). The ECCO‐Darwin data‐assimilative global ocean biogeochemistry model: Estimates of seasonal to multidecadal Surface Ocean pCO_2_ and air‐sea CO_2_ flux. Journal of Advances in Modeling Earth Systems, 12(10), 1–28. 10.1029/2019ms001888

[gbc21250-bib-0012] Chatterjee, A. , Gierach, M. M. , Sutton, A. J. , Feely, R. A. , Crisp, D. , Eldering, A. , et al. (2017). Influence of El Niño on atmospheric CO_2_ over the tropical Pacific Ocean: Findings from NASA's OCO‐2 mission. Science, 358(6360). 10.1126/science.aam5776 PMC5668685 29026014

[gbc21250-bib-0013] Chen, C.‐T. A. , Huang, T.‐H. , Chen, Y.‐C. , Bai, Y. , He, X. , & Kang, Y. (2013). Air–sea exchanges of CO_2_ in the world’s coastal seas. Biogeosciences, 10(10), 6509–6544. 10.5194/bg-10-6509-2013

[gbc21250-bib-0014] Crisp, D. , Pollock, H. R. , Rosenberg, R. , Chapsky, L. , Lee, R. A. M. , Oyafuso, F. A. , et al. (2017). The on‐orbit performance of the Orbiting Carbon Observatory‐2 (OCO‐2) instrument and its radiometrically calibrated products. Atmospheric Measurement Techniques, 10(1), 59–81. 10.5194/amt-10-59-2017

[gbc21250-bib-0015] DeVries, T. (2014). The oceanic anthropogenic CO_2_ sink: Storage, air‐sea fluxes, and transports over the industrial era. Global Biogeochemical Cycles, 28(7), 631–647. 10.1002/2013gb004739

[gbc21250-bib-0016] DeVries, T. , Holzer, M. , & Primeau, F. (2017). Recent increase in oceanic carbon uptake driven by weaker upper‐ocean overturning. Nature, 542(7640), 214–218. 10.1038/nature21068 28179663

[gbc21250-bib-0017] DeVries, T. , Le Quéré, C. , Andrews, O. , Berthet, S. , Hauck, J. , Ilyina, T. , et al. (2019). Decadal trends in the ocean carbon sink. Proceedings of the National Academy of Sciences, 116(24), 11646–11651. 10.1073/pnas.1900371116 PMC6576185 31138699

[gbc21250-bib-0018] DeVries, T. , & Weber, T. (2017). The export and fate of organic matter in the ocean: New constraints from combining satellite and oceanographic tracer observations. Global Biogeochemical Cycles, 31(3), 535–555. 10.1002/2016gb005551

[gbc21250-bib-0019] Dunne, J. P. (2016). Quantifying uncertainty in future ocean carbon uptake. Global Biogeochemical Cycles, 30(1), 1563–1565. 10.1002/2016gb005525

[gbc21250-bib-0020] Dunne, J. P. , Sarmiento, J. L. , & Gnanadesikan, A. (2007). A synthesis of global particle export from the surface ocean and cycling through the ocean interior and on the seafloor. Global Biogeochemical Cycles, 21(4), GB4006. 10.1029/2006gb002907

[gbc21250-bib-0021] Dutkiewicz, S. , Cermeno, P. , Jahn, O. , Follows, M. J. , Hickman, A. E. , Taniguchi, D. A. A. , & Ward, B. A. (2020). Dimensions of marine phytoplankton diversity. Biogeosciences, 17(3), 609–634. 10.5194/bg-17-609-2020

[gbc21250-bib-0022] Dutkiewicz, S. , Hickman, A. E. , & Jahn, O. (2018). Modelling ocean‐colour‐derived chlorophyll *a* . Biogeosciences, 15(2), 613–630. 10.5194/bg-15-613-2018

[gbc21250-bib-0023] Dutkiewicz, S. , Hickman, A. E. , Jahn, O. , Gregg, W. W. , Mouw, C. B. , & Follows, M. J. (2015). Capturing optically important constituents and properties in a marine biogeochemical and ecosystem model. Biogeosciences, 12(14), 4447–4481. 10.5194/bg-12-4447-2015

[gbc21250-bib-0024] Dutkiewicz, S. , Hickman, A. E. , Jahn, O. , Henson, S. , Beaulieu, C. , & Monier, E. (2019). Ocean colour signature of climate change. Nature Communications, 10(1), 578. 10.1038/s41467-019-08457-x PMC6362115 30718491

[gbc21250-bib-0025] Estapa, M. L. , Feen, M. L. , & Breves, E. (2019). Direct observations of biological carbon export from profiling floats in the subtropical North Atlantic. Global Biogeochemical Cycles, 33(3), 282–300. 10.1029/2018gb006098

[gbc21250-bib-0026] Falkowski, P. , Scholes, R. J. , Boyle, E. , Canadell, J. , Canfield, D. , Elser, J. , et al. (2000). The global carbon cycle: A test of our knowledge of Earth as a system. Science, 290(5490), 291–296. 10.1126/science.290.5490.291 11030643

[gbc21250-bib-0027] Fay, A. R. , & McKinley, G. A. (2014). Global open‐ocean biomes: Mean and temporal variability. Earth System Science Data, 6(2), 273–284. 10.5194/essd-6-273-2014

[gbc21250-bib-0028] Fay, A. R. , McKinley, G. A. , & Lovenduski, N. S. (2014). Southern Ocean carbon trends: Sensitivity to methods. Geophysical Research Letters, 41(19), 6833–6840. 10.1002/2014gl061324

[gbc21250-bib-0029] Feely, R. A. , Boutin, J. , Cosca, C. E. , Dandonneau, Y. , Etcheto, J. , Inoue, H. Y. , et al. (2002). Seasonal and interannual variability of CO_2_ in the equatorial Pacific. Deep Sea Research Part II: Topical Studies in Oceanography, 49(13), 2443–2469. 10.1016/s0967-0645(02)00044-9

[gbc21250-bib-0030] Fekete, B. M. , Vörösmarty, C. J. , & Grabs, W. (2002). High‐resolution fields of global runoff combining observed river discharge and simulated water balances. Global Biogeochemical Cycles, 16(3), 15‐1–15‐10. 10.1029/1999gb001254

[gbc21250-bib-0031] Feng, Y. , Menemenlis, D. , Xue, H. , Zhang, H. , Carroll, D. , Du, Y. , & Wu, H. (2021). Improved representation of river runoff in Estimating the Circulation and Climate of the Ocean version 4 (eccov4) simulations: Implementation, evaluation, and impacts to coastal plume regions. Geoscientific Model Development, 14(3), 1801–1819. 10.5194/gmd-14-1801-2021

[gbc21250-bib-0032] Follows, M. J. , Dutkiewicz, S. , Grant, S. , & Chisholm, S. W. (2007). Emergent biogeography of microbial communities in a model ocean. Science, 315(5820), 1843–1846. 10.1126/science.1138544 17395828

[gbc21250-bib-0033] Follows, M. J. , Ito, T. , & Dutkiewicz, S. (2006). On the solution of the carbonate chemistry system in ocean biogeochemistry models. Ocean Modelling, 12(3), 290–301. 10.1016/j.ocemod.2005.05.004

[gbc21250-bib-0034] Friedlingstein, P. , Jones, M. W. , O’Sullivan, M. , Andrew, R. M. , Hauck, J. , Peters, G. P. , et al. (2019). Global carbon budget 2019. Earth System Science Data, 11(4), 1783–1838. 10.5194/essd-11-1783-2019

[gbc21250-bib-0035] Gaspar, P. , Grégoris, Y. , & Lefevre, J.‐M. (1990). A simple eddy kinetic energy model for simulations of the oceanic vertical mixing: Tests at station papa and long‐term upper ocean study site. Journal of Geophysical Research: Oceans, 95(C9), 16179–16193. 10.1029/jc095ic09p16179

[gbc21250-bib-0036] Gent, P. R. , & McWilliams, J. C. (1990). Isopycnal mixing in ocean circulation models. Journal of Physical Oceanography, 20(1), 150–155. 10.1175/1520-0485(1990)020<0150:imiocm>2.0.co;2

[gbc21250-bib-0037] Gent, P. R. , Willebrand, J. , McDougall, T. J. , & McWilliams, J. C. (1995). Parameterizing eddy‐induced tracer transports in ocean circulation models. Journal of Physical Oceanography, 25(4), 463–474. 10.1175/1520-0485(1995)025<0463:peitti>2.0.co;2

[gbc21250-bib-0038] Gierach, M. M. , Lee, T. , Turk, D. , & McPhaden, M. J. (2012). Biological response to the 1997–98 and 2009–10 El Niño events in the equatorial Pacific Ocean. Geophysical Research Letters, 39(10). 10.1029/2012gl051103

[gbc21250-bib-0039] Gloege, L. , McKinley, G. A. , Landschützer, P. , Fay, A. R. , Frölicher, T. L. , Fyfe, J. C. , et al. (2021). Quantifying errors in observationally based estimates of ocean carbon sink variability. Global Biogeochemical Cycles, 35(4), e2020GB006788. 10.1029/2020gb006788

[gbc21250-bib-0040] Gregor, L. , & Gruber, N. (2021). OceanSODA‐ETHZ: A global gridded data set of the Surface Ocean carbonate system for seasonal to decadal studies of ocean acidification. Earth System Science Data, 13(2), 777–808. 10.5194/essd-13-777-2021

[gbc21250-bib-0041] Gruber, N. , Clement, D. , Carter, B. R. , Feely, R. A. , van Heuven, S. , Hoppema, M. , et al. (2019). The oceanic sink for anthropogenic CO_2_ from 1994 to 2007. Science, 363(6432), 1193–1199. 10.1126/science.aau5153 30872519

[gbc21250-bib-0042] Hansell, D. A. (2013). Recalcitrant dissolved organic carbon fractions. Annual Review of Marine Science, 5(1), 421–445. 10.1146/annurev-marine-120710-100757 22881353

[gbc21250-bib-0043] Heinze, C. , Meyer, S. , Goris, N. , Anderson, L. , Steinfeldt, R. , Chang, N. , et al. (2015). The ocean carbon sink – Impacts, vulnerabilities and challenges. Earth System Dynamics, 6(1), 327–358. 10.5194/esd-6-327-2015

[gbc21250-bib-0044] Hopwood, M. J. , Carroll, D. , Browning, T. J. , Meire, L. , Mortensen, J. , Krisch, S. , & Achterberg, E. P. (2018). Non‐linear response of summertime marine productivity to increased meltwater discharge around Greenland. Nature Communications, 9(3256). 10.1038/s41467-018-05488-8 PMC6092443 30108210

[gbc21250-bib-0045] Hopwood, M. J. , Carroll, D. , Dunse, T. , Hodson, A. , Holding, J. M. , Iriarte, J. L. , et al. (2020). Review article: How does glacier discharge affect marine biogeochemistry and primary production in the arctic? The Cryosphere, 14(4), 1347–1383. 10.5194/tc-14-1347-2020

[gbc21250-bib-0046] IPCC . (2013). Summary for policymakers [book section]. In T. Stocker et al. (Eds.), In Climate change 2013: The physical science basis. contribution of Working group I to the Fifth assessment report of the Intergovernmental Panel on Climate change. 1–30. Cambridge University Press.

[gbc21250-bib-0047] Keppler, L. , Landschützer, P. , Gruber, N. , Lauvset, S. K. , & Stemmler, I. (2020). Seasonal carbon dynamics in the near‐global ocean. Global Biogeochemical Cycles, 34(12), e2020GB006571. 10.1029/2020gb006571

[gbc21250-bib-0048] Khatiwala, S. , Primeau, F. , & Hall, T. (2009). Reconstruction of the history of anthropogenic CO_2_ concentrations in the ocean. Nature, 462(7271), 346–349. 10.1038/nature08526 19924213

[gbc21250-bib-0049] Khatiwala, S. , Tanhua, T. , Mikaloff Fletcher, S. , Gerber, M. , Doney, S. C. , Graven, H. D. , et al. (2013). Global ocean storage of anthropogenic carbon. Biogeosciences, 10(4), 2169–2191. 10.5194/bg-10-2169-2013

[gbc21250-bib-0050] Landschützer, P. , Gruber, N. , & Bakker, D. C. E. (2016). Decadal variations and trends of the global ocean carbon sink. Global Biogeochemical Cycles, 30(10), 1396–1417. 10.1002/2015gb005359

[gbc21250-bib-0051] Landschützer, P. , Gruber, N. , Bakker, D. C. E. , Schuster, U. , Nakaoka, S. , Payne, M. R. , et al. (2013). A neural network‐based estimate of the seasonal to inter‐annual variability of the Atlantic Ocean carbon sink. Biogeosciences, 10(11), 7793–7815. 10.5194/bg-10-7793-2013

[gbc21250-bib-0052] Lauderdale, J. M. , Dutkiewicz, S. , Williams, R. G. , & Follows, M. J. (2016). Quantifying the drivers of ocean‐atmosphere CO_2_ fluxes. Global Biogeochemical Cycles, 30(7), 983–999. 10.1002/2016GB005400

[gbc21250-bib-0053] Lauvset, S. K. , Key, R. M. , Olsen, A. , van Heuven, S. , Velo, A. , Lin, X. , et al. (2016). A new global interior ocean mapped climatology: The 1° × 1° GLODAP version 2. Earth System Science Data, 8(2), 325–340. 10.5194/essd-8-325-2016

[gbc21250-bib-0054] Lauvset, S. K. , Lange, N. , Tanhua, T. , Bittig, H. C. , Olsen, A. , Kozyr, A. , et al. (2021). An updated version of the global interior ocean biogeochemical data product, GLODAPv2.2021. Earth System Science Data Discussions, 13(12), 1–32. 10.5194/essd-13-5565-2021

[gbc21250-bib-0055] Lee, K. (2001). Global net community production estimated from the annual cycle of surface water total dissolved inorganic carbon. Limnology & Oceanography, 46(6), 1287–1297. 10.4319/lo.2001.46.6.1287

[gbc21250-bib-0056] Le Fouest, V. , Babin, M. , & Tremblay, J.‐E. (2013). The fate of riverine nutrients on arctic shelves. Biogeosciences, 10(6), 3661–3677. 10.5194/bg-10-3661-2013

[gbc21250-bib-0057] Le Fouest, V. , Manizza, M. , Tremblay, B. , & Babin, M. (2015). Modelling the impact of riverine don removal by marine bacterioplankton on primary production in the arctic ocean. Biogeosciences, 12(11), 3385–3402. 10.5194/bg-12-3385-2015

[gbc21250-bib-0058] Le Quéré, C. , Andrew, R. M. , Friedlingstein, P. , Sitch, S. , Hauck, J. , Pongratz, J. , et al. (2018). Global Carbon Budget 2018. Earth System Science Data, 10(4), 2141–2194. 10.5194/essd-10-2141-2018

[gbc21250-bib-0059] Li, Z. , & Cassar, N. (2016). Satellite estimates of net community production based on O_2_/Ar observations and comparison to other estimates. Global Biogeochemical Cycles, 30(5), 735–752. 10.1002/2015gb005314

[gbc21250-bib-0060] Liu, J. , Baskaran, L. , Bowman, K. , Schimel, D. , Bloom, A. A. , Parazoo, N. C. , et al. (2021). Carbon monitoring system flux net biosphere exchange 2020 (CMS‐flux NBE 2020). Earth System Science Data, 13(2), 299–330. 10.5194/essd-13-299-2021

[gbc21250-bib-0061] Liu, J. , Bowman, K. W. , Lee, M. , Henze, D. K. , Bousserez, N. , Brix, H. , et al. (2014). Carbon monitoring system flux estimation and attribution: Impact of ACOS‐GOSAT XCO_2_ sampling on the inference of terrestrial biospheric sources and sinks. Tellus B: Chemical and Physical Meteorology, 66(1), 22486. 10.3402/tellusb.v66.22486

[gbc21250-bib-0062] Liu, J. , Bowman, K. W. , Schimel, D. S. , Parazoo, N. C. , Jiang, Z. , Lee, M. , et al. (2017). Contrasting carbon cycle responses of the tropical continents to the 2015—2016 El Niño. Science, 358(6360), eaam5690. 10.1126/science.aam5690 29026011

[gbc21250-bib-0063] Lovenduski, N. S. , Long, M. C. , Gent, P. R. , & Lindsay, K. (2013). Multi‐decadal trends in the advection and mixing of natural carbon in the Southern Ocean. Geophysical Research Letters, 40(1), 139–142. 10.1029/2012gl054483

[gbc21250-bib-0064] Lozier, M. S. , Dave, A. C. , Palter, J. B. , Gerber, L. M. , & Barber, R. T. (2011). On the relationship between stratification and primary productivity in the North Atlantic. Geophysical Research Letters, 38(18). 10.1029/2011gl049414

[gbc21250-bib-0065] Mahadevan, A. (2016). The impact of submesoscale physics on primary productivity of plankton. Annual Review of Marine Science, 8(1), 161–184. 10.1146/annurev-marine-010814-015912 26394203

[gbc21250-bib-0066] Mahowald, N. M. , Engelstaedter, S. , Luo, C. , Sealy, A. , Artaxo, P. , Benitez‐Nelson, C. , et al. (2009). Atmospheric iron deposition: Global distribution, variability, and human perturbations. Annual Review of Marine Science, 1(1), 245–278. 10.1146/annurev.marine.010908.163727 21141037

[gbc21250-bib-0067] Manizza, M. , Menemenlis, D. , Zhang, H. , & Miller, C. E. (2019). Modeling the recent changes in the Arctic Ocean CO_2_ sink (2006–2013). Global Biogeochemical Cycles, 33(3), 420–438. 10.1029/2018gb006070

[gbc21250-bib-0068] Mayorga, E. , Seitzinger, S. P. , Harrison, J. A. , Dumont, E. , Beusen, A. H. , Bouwman, A. , et al. (2010). Global nutrient export from WaterSheds 2 (NEWS 2): Model development and implementation. Environmental Modelling & Software, 25(7), 837–853. 10.1016/j.envsoft.2010.01.007

[gbc21250-bib-0069] McKinley, G. A. , Fay, A. R. , Eddebbar, Y. A. , Gloege, L. , & Lovenduski, N. S. (2020). External forcing explains recent decadal variability of the ocean carbon sink. AGU Advances, 1(2), e2019AV000149. 10.1029/2019av000149

[gbc21250-bib-0070] McKinley, G. A. , Fay, A. R. , Lovenduski, N. S. , & Pilcher, D. J. (2017). Natural variability and anthropogenic trends in the ocean carbon sink. Annual Review of Marine Science, 9(1), 125–150. 10.1146/annurev-marine-010816-060529 27620831

[gbc21250-bib-0071] McKinley, G. A. , Follows, M. J. , & Marshall, J. (2004). Mechanisms of air‐sea CO_2_ flux variability in the equatorial Pacific and the North Atlantic. Global Biogeochemical Cycles, 18(2), GB2011. 10.1029/2003gb002179

[gbc21250-bib-0072] Menemenlis, D. , Fukumori, I. , & Lee, T. (2005). Using Green’s Functions to calibrate an ocean general circulation model. Monthly Weather Review, 133(5), 1224–1240. 10.1175/mwr2912.1

[gbc21250-bib-0073] Olsen, A. , Lange, N. , Key, R. M. , Tanhua, T. , Bittig, H. C. , Kozyr, A. , et al. (2020). An updated version of the global interior ocean biogeochemical data product, GLODAPv2.2020. Earth System Science Data, 12(4), 3653–3678. 10.5194/essd-12-3653-2020

[gbc21250-bib-0074] Ott, L. E. , Pawson, S. , Collatz, G. J. , Gregg, W. W. , Menemenlis, D. , Brix, H. , et al. (2015). Assessing the magnitude of CO_2_ flux uncertainty in atmospheric CO_2_ records using products from NASA’s Carbon Monitoring Flux Pilot Project. Journal of Geophysical Research: Atmospheres, 120(2), 734–765. 10.1002/2014jd022411

[gbc21250-bib-0075] Patra, P. K. , Crisp, D. , Kaiser, J. W. , Wunch, D. , Saeki, T. , Ichii, K. , et al. (2017). The Orbiting Carbon Observatory (OCO‐2) tracks 2–3 peta‐gram increase in carbon release to the atmosphere during the 2014–2016 el niño. Scientific Reports, 7(1), 13567. 10.1038/s41598-017-13459-0 29051612 PMC5648889

[gbc21250-bib-0076] Quetin, G. R. , Bloom, A. A. , Bowman, K. W. , & Konings, A. G. (2020). Carbon flux variability from a relatively simple ecosystem model with assimilated data is consistent with terrestrial biosphere model estimates. Journal of Advances in Modeling Earth Systems, 12(3), e2019MS001889. 10.1029/2019ms001889

[gbc21250-bib-0077] Randerson, J. T. , Lindsay, K. , Munoz, E. , Fu, W. , Moore, J. K. , Hoffman, F. M. , et al. (2015). Multicentury changes in ocean and land contributions to the climate‐carbon feedback. Global Biogeochemical Cycles, 29(6), 744–759. 10.1002/2014gb005079

[gbc21250-bib-0078] Redi, M. H. (1982). Oceanic isopycnal mixing by coordinate rotation. Journal of Physical Oceanography, 12(10), 1154–1158. 10.1175/1520-0485(1982)012<1154:oimbcr>2.0.co;2

[gbc21250-bib-0079] Resplandy, L. , Keeling, R. F. , Rödenbeck, C. , Stephens, B. , Khatiwala, S. , Rodgers, K. B. , et al. (2018). Revision of global carbon fluxes based on a reassessment of oceanic and riverine carbon transport. Nature Geoscience, 11(7), 504–509. 10.1038/s41561-018-0151-3

[gbc21250-bib-0080] Resplandy, L. , Séférian, R. , & Bopp, L. (2015). Natural variability of CO_2_ and O_2_ fluxes: What can we learn from centuries‐long climate models simulations? Journal of Geophysical Research: Oceans, 120(1), 384–404. 10.1002/2014jc010463

[gbc21250-bib-0081] Ritter, R. , Landschützer, P. , Gruber, N. , Fay, A. R. , Iida, Y. , Jones, S. , et al. (2017). Observation‐based trends of the Southern Ocean carbon sink. Geophysical Research Letters, 44(2412), 12339–12348. 10.1002/2017gl074837

[gbc21250-bib-0082] Rödenbeck, C. , Bakker, D. C. E. , Gruber, N. , Iida, Y. , Jacobson, A. R. , Jones, S. , et al. (2015). Data‐based estimates of the ocean carbon sink variability – First results of the surface ocean pco_2_ mapping intercomparison (socom). Biogeosciences, 12(23), 7251–7278. 10.5194/bg-12-7251-2015

[gbc21250-bib-0083] Rödenbeck, C. , Keeling, R. F. , Bakker, D. C. E. , Metzl, N. , Olsen, A. , Sabine, C. , & Heimann, M. (2013). Global surface‐ocean pCO_2_ and sea‐air CO_2_ flux variability from an observation‐driven ocean mixed‐layer scheme. Ocean Science, 9(2), 193–216. 10.5194/os-9-193-2013

[gbc21250-bib-0084] Rosso, I. , Mazloff, M. R. , Verdy, A. , & Talley, L. D. (2017). Space and time variability of the Southern Ocean carbon budget. Journal of Geophysical Research: Oceans, 122(9), 7407–7432. 10.1002/2016jc012646

[gbc21250-bib-0085] Sabine, C. L. , Feely, R. A. , Gruber, N. , Key, R. M. , Lee, K. , Bullister, J. L. , et al. (2004). The oceanic sink for anthropogenic CO_2_ . Science, 305(5682), 367–371. 10.1126/science.1097403 15256665

[gbc21250-bib-0086] Sarmiento, J. L. , & Gruber, N. (2002). Sinks for anthropogenic carbon. Physics Today, 55(8), 30–36. 10.1063/1.1510279

[gbc21250-bib-0087] Sarmiento, J. L. , & Gruber, N. (2006). Carbon cycle, CO_2_, and climate; the anthropogenic perturbation, ocean biogeochemical dynamics. Princeton University Press.

[gbc21250-bib-0088] Siegel, D. A. , Buesseler, K. O. , Behrenfeld, M. J. , Benitez‐Nelson, C. R. , Boss, E. , Brzezinski, M. A. , et al. (2016). Prediction of the export and fate of global ocean net primary production: The EXPORTS science plan. Frontiers in Marine Science, 3. 10.3389/fmars.2016.00022

[gbc21250-bib-0089] Silvano, A. (2020). Changes in the Southern Ocean. Nature Geoscience, 13(1), 4–5. 10.1038/s41561-019-0516-2

[gbc21250-bib-0090] Stock, C. A. , Dunne, J. P. , & John, J. G. (2014). Global‐scale carbon and energy flows through the marine planktonic food web: An analysis with a coupled physical–biological model. Progress in Oceanography, 120, 1–28. 10.1016/j.pocean.2013.07.001

[gbc21250-bib-0091] Sulpis, O. , Humphreys, M. , Wilhelmus, M. , Carroll, D. , Berelson, W. , Menemenlis, D. , & Adkins, J. (2021). RADIv1: A non‐steady‐state early diagenetic model for ocean sediments in Julia and MATLAB/GNU Octave. Geoscientific Model Development, 15, 2105–2131. 10.5194/gmd-15-2105-2022

[gbc21250-bib-0092] Sutton, A. J. , Feely, R. A. , Sabine, C. L. , McPhaden, M. J. , Takahashi, T. , Chavez, F. P. , et al. (2014). Natural variability and anthropogenic change in equatorial Pacific surface ocean pCO_2_ and pH. Global Biogeochemical Cycles, 28(2), 131–145. 10.1002/2013gb004679

[gbc21250-bib-0093] Takahashi, T. , Sutherland, S. C. , Sweeney, C. , Poisson, A. , Metzl, N. , Tilbrook, B. , et al. (2002). Global sea–air CO_2_ flux based on climatological surface ocean pCO_2_, and seasonal biological and temperature effects. Deep Sea Research Part II: Topical Studies in Oceanography, 49(9), 1601–1622. 10.1016/s0967-0645(02)00003-6

[gbc21250-bib-0094] Talley, L. D. (2013). Closure of the global overturning circulation through the Indian, Pacific, and Southern Oceans: Schematics and transports. Oceanography, 26(1), 80–97. 10.5670/oceanog.2013.07

[gbc21250-bib-0095] Verdy, A. , & Mazloff, M. R. (2017). A data assimilating model for estimating Southern Ocean biogeochemistry. Journal of Geophysical Research: Oceans, 122(9), 6968–6988. 10.1002/2016jc012650

[gbc21250-bib-0096] Wadham, J. L. , Hawkings, J. R. , Tarasov, L. , Gregoire, L. J. , Spencer, R. G. M. , Gutjahr, M. , et al. (2019). Ice sheets matter for the global carbon cycle. Nature Communications, 10(1), 3567. 10.1038/s41467-019-11394-4 PMC6695407 31417076

[gbc21250-bib-0097] Wanninkhof, R. (1992). Relationship between wind speed and gas exchange over the ocean. Journal of Geophysical Research: Oceans, 97(C5), 7373–7382. 10.1029/92jc00188

[gbc21250-bib-0098] Wanninkhof, R. , Park, G. H. , Takahashi, T. , Sweeney, C. , Feely, R. , Nojiri, Y. , et al. (2013). Global ocean carbon uptake: Magnitude, variability and trends. Biogeosciences, 10(3), 1983–2000. 10.5194/bg-10-1983-2013

[gbc21250-bib-0099] Whitt, D. B. , Lévy, M. , & Taylor, J. R. (2019). Submesoscales enhance storm‐driven vertical mixing of nutrients: Insights from a biogeochemical large eddy simulation. Journal of Geophysical Research: Oceans, 124(11), 8140–8165. 10.1029/2019jc015370

[gbc21250-bib-0100] Wunsch, C. , & Heimbach, P. (2013). Dynamically and kinematically consistent global ocean circulation and ice state estimates. In G. Siedler , S. M. Griffies , J. Gould , & J. A. Church (Eds.), Ocean circulation and climate: A 21st century perspect (Vol. 103, pp. 553–579). Academic Press.

[gbc21250-bib-0101] Wunsch, C. , Heimbach, P. , Ponte, R. , & Fukumori, I. (2009). The global general circulation of the ocean estimated by the ECCO‐consortium. Oceanography, 22(2), 88–103. 10.5670/oceanog.2009.41

[gbc21250-bib-0102] Zhang, H. , Menemenlis, D. , & Fenty, I. G. (2018). ECCO LLC270 ocean‐ice state estimate (Tech. Rep.). Jet Propulsion Laboratory. California Institute of Technology. Available at: http://hdl.handle.net/1721.1/119821

