# Supplementary material for: Attribution of Space‐Time Variability in Global‐Ocean Dissolved Inorganic Carbon
Source: Global Biogeochem Cycles. 2022 Mar 22;36(3):e2021GB007162. doi: 10.1029/2021GB007162 (PMC9286438; doi:10.1029/2021GB007162)
Supplement: Supplementary file 1 — Supporting Information S1 [file GBC-36-0-s001.pdf]

Supporting Information for "Attribution of space-time variability in global-ocean dissolved inorganic carbon"

Dustin Carroll[1,2], Dimitris Menemenlis[2], Stephanie Dutkiewicz[3,4], Jonathan Lauderdale[3], Jess F. Adkins[5], Kevin W. Bowman[2], Holger Brix[6,7], Ian Fenty[2], Michelle M. Gierach[2], Chris Hill[3], Oliver Jahn[3], Peter Landschützer[8], Manfredi Manizza[9], Matt R. Mazloff[9], Charles E. Miller[2], David S. Schimel[2], Ariane Verdy[9], Daniel B. Whitt[10], Hong Zhang[2]

[1]Moss Landing Marine Laboratories, San José State University, Moss Landing, CA, USA

[2]Jet Propulsion Laboratory, California Institute of Technology, Pasadena, CA, USA

[3]Department of Earth, Atmospheric and Planetary Sciences, Massachusetts Institute of Technology, Cambridge, Massachusetts, USA

[4]Center for Global Change Science, Massachusetts Institute of Technology, Cambridge, Massachusetts, USA

[5]Division of Geological and Planetary Sciences, California Institute of Technology, Pasadena, California, USA

[6]Joint Institute for Regional Earth System Science and Engineering, University of California Los Angeles. Los Angeles, CA, USA

[7]Institute of Coastal Ocean Dynamics, Helmholtz-Zentrum Hereon, Geesthacht, Germany

[8]Max Planck Institute for Meteorology, Hamburg, Germany

[9]Scripps Institution of Oceanography, University of California San Diego, La Jolla, California, USA

[10]NASA Ames Research Center, Moffett Field, CA, USA

Contents of this file

1. Text S1
2. Figures S1 to S7
3. Tables S1 to S2

Text S1.

In the z* rescaled vertical coordinates of Campin et al. (2008), the thickness of vertical levels are scaled proportionally with sea-surface height divided by depth. Specifically, the z* scaling factor is sea-surface height times nominal level thickness divided by nominal model depth at each horizontal location. Consider 1-m water equivalent of sea ice on top of a model surface level of 10 m (*L1*) and a set of deeper model levels 1000-m thick (*L2*). The concentration of DIC in both of these levels is c. The mass of DIC in *L1* and *L2* is proportional to $10c$ and $1000c$, respectively. If you melt the 1 m of frozen water, assuming that sea ice does not contain any DIC, the thickness of the levels will be increased by a scaling factor of 1/1010, that is *L1* = 10.01 m and *L2* = 1000.99 m, respectively. To a good approximation, the concentration of DIC in *L1* and *L2* will be $0.9c$ and c, respectively. Therefore the mass of DIC in *L1* and *L2* will be proportional to $9c$ and $1001c$, respectively. That is, the process of surface-level dilution due to sea-ice melt in z* coordinates is equivalent to redistributing $1c$ mass equivalent of DIC from the

---

Corresponding author: D. Carroll, Moss Landing Marine Laboratories, San José State University, Moss Landing, CA, USA. (dustin.carroll@sjsu.edu)

surface level to the levels below. Conversely, sea-ice formation will redistribute DIC from deeper levels to the surface level.

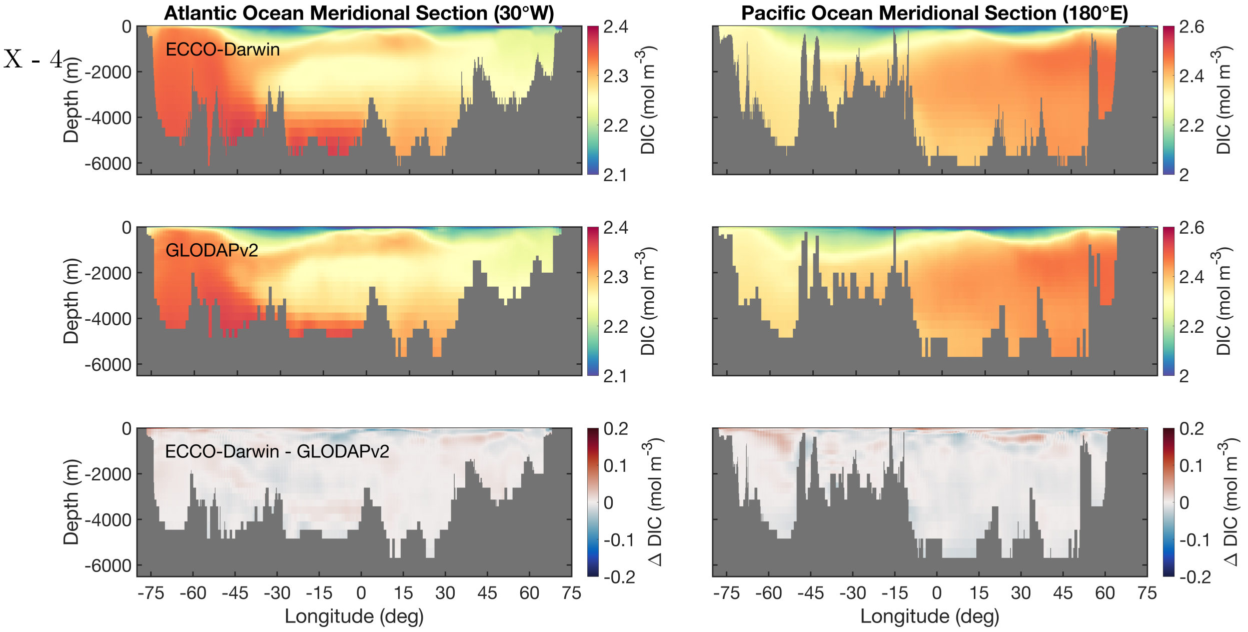

Figure S1. Comparison of climatological ECCO-Darwin DIC (1995–2018) and the GLODAPv2 mapped product for Pacific and Atlantic Ocean meridional sections.

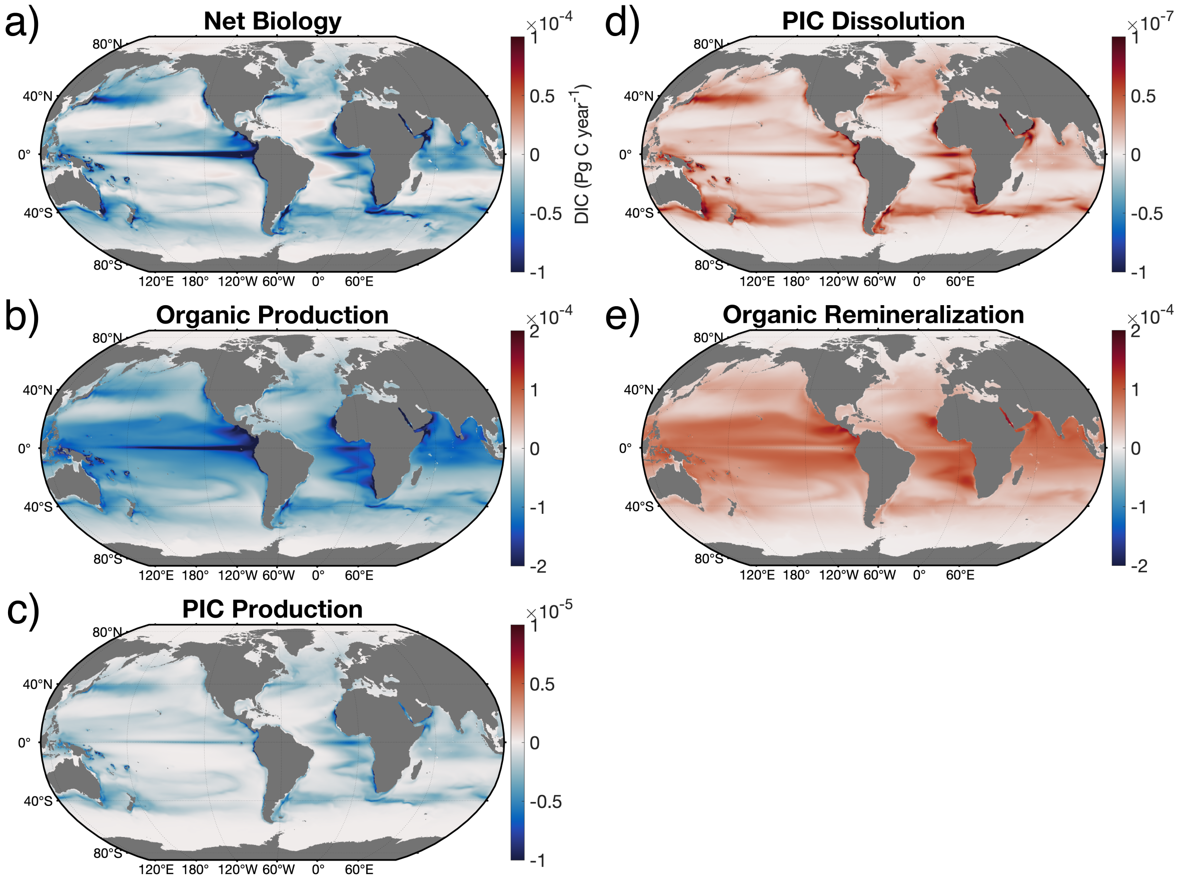

Figure S2. Climatology of mass budget biology terms, vertically integrated from the surface to 100-m depth. Positive values (red colors) represent DIC gain and negative values (blue colors) show loss. The net biology term shown in **a)** is the sum of panels **b–e)**. Note the different scales used.

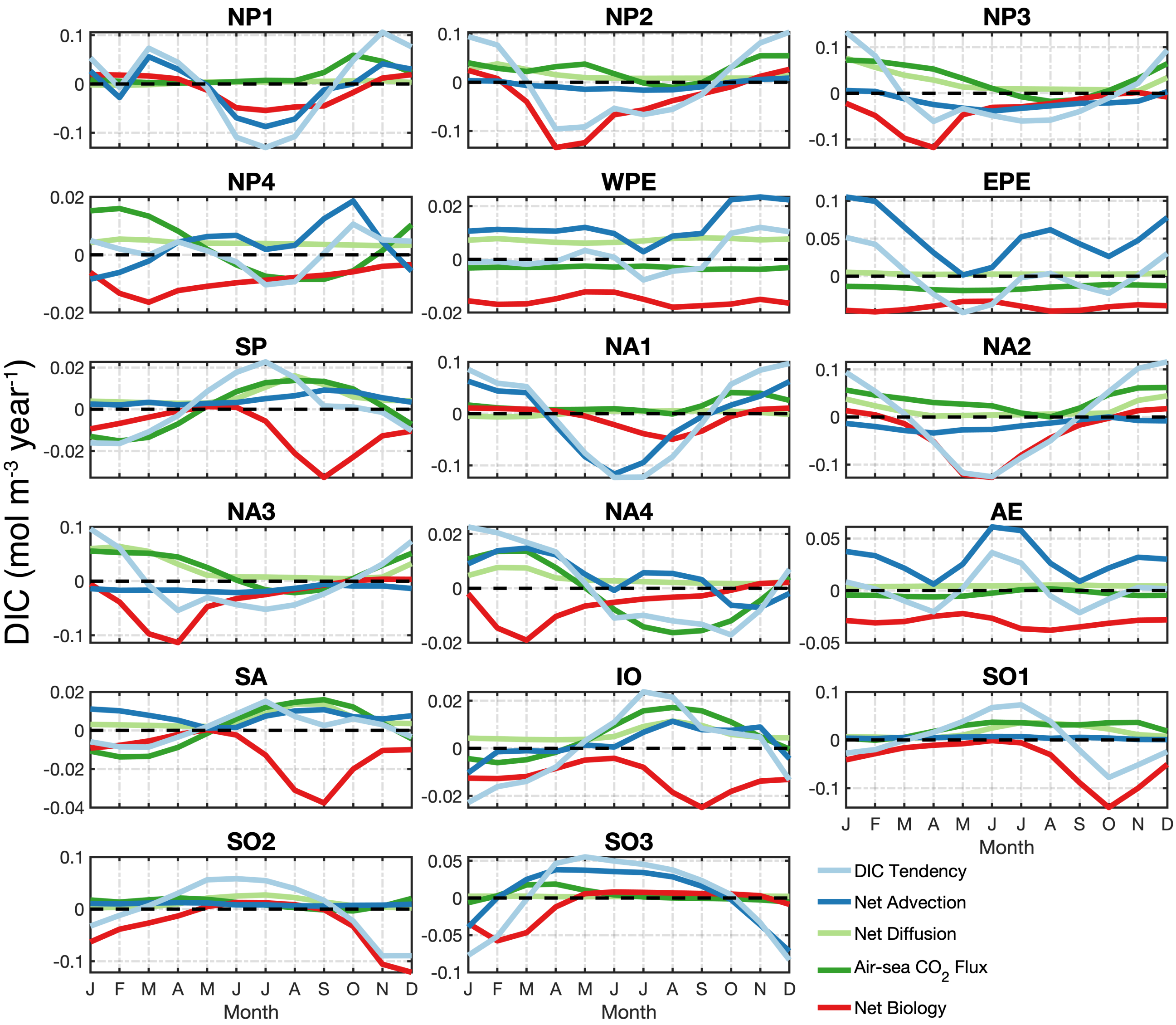

Figure S3. Seasonal climatology of concentration budget terms in the upper-100 m for all biomes; dashed black lines show the zero value.

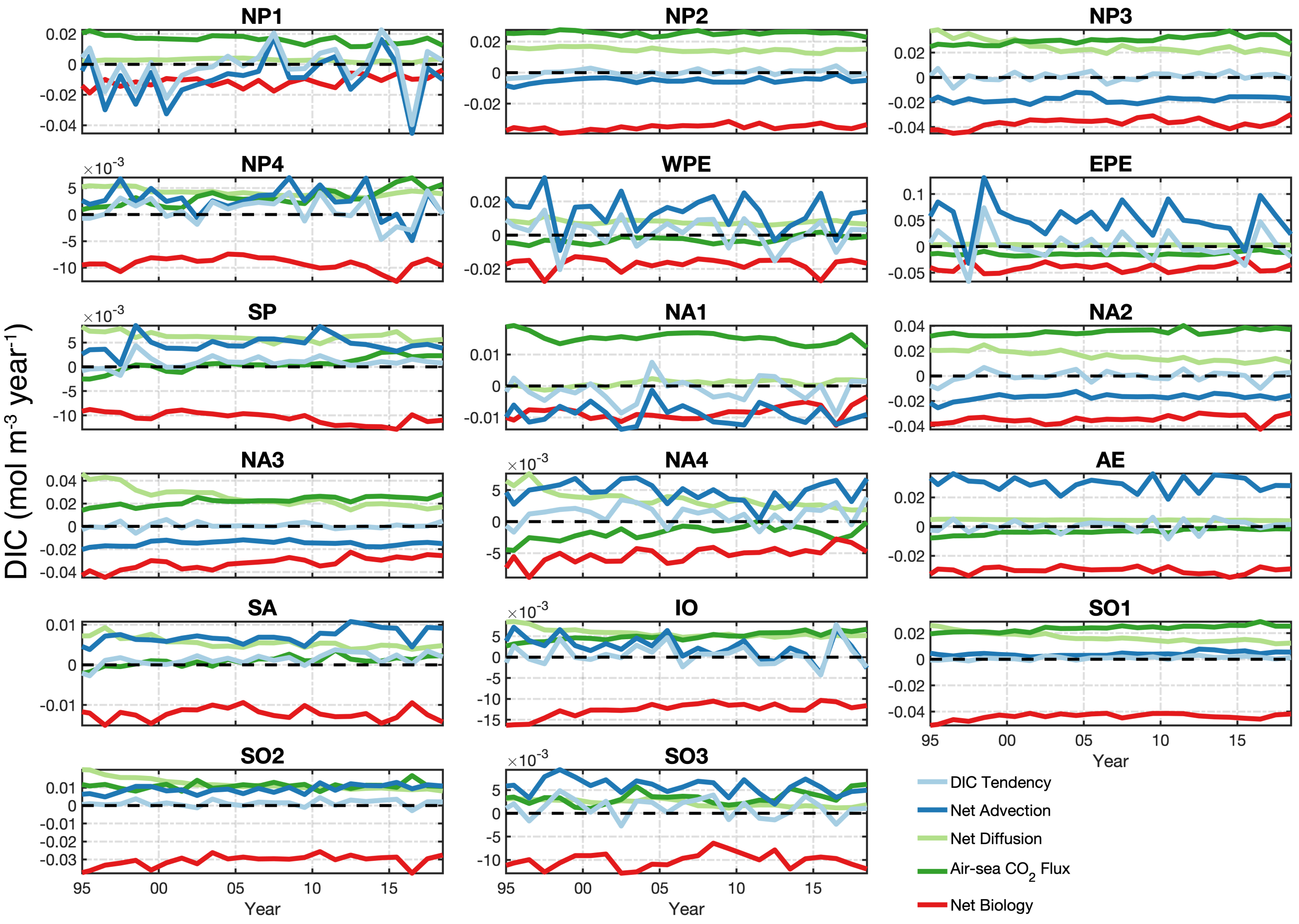

Figure S4. Annual-mean time series of concentration budget terms in the upper-100 m for all biomes; dashed black lines show the zero value.

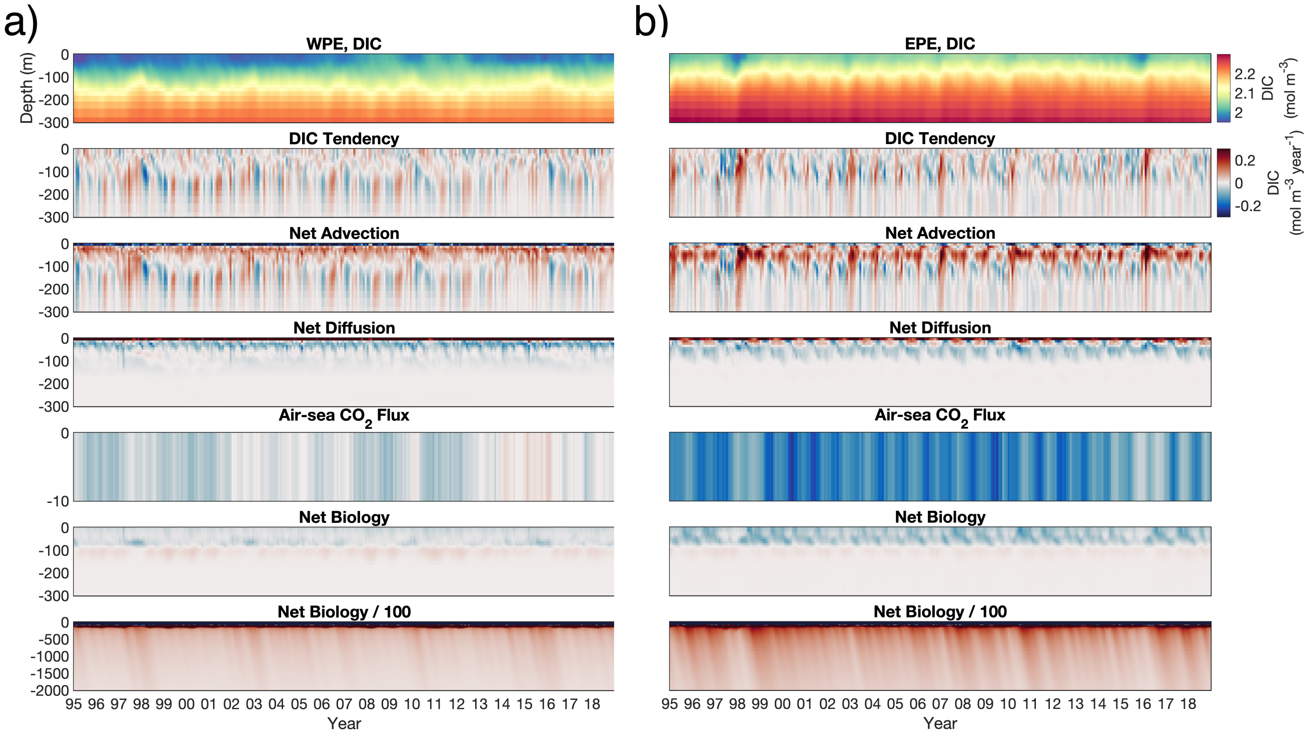

Figure S5. Monthly-mean Hovmöller diagram of upper-ocean DIC concentration and associated budget terms in equatorial Pacific Ocean biomes **a)** WPE and **b)** EPE. Bottom row shows net biology for the upper-2000-m water column; here values are scaled by a factor of 100 to increase visibility.

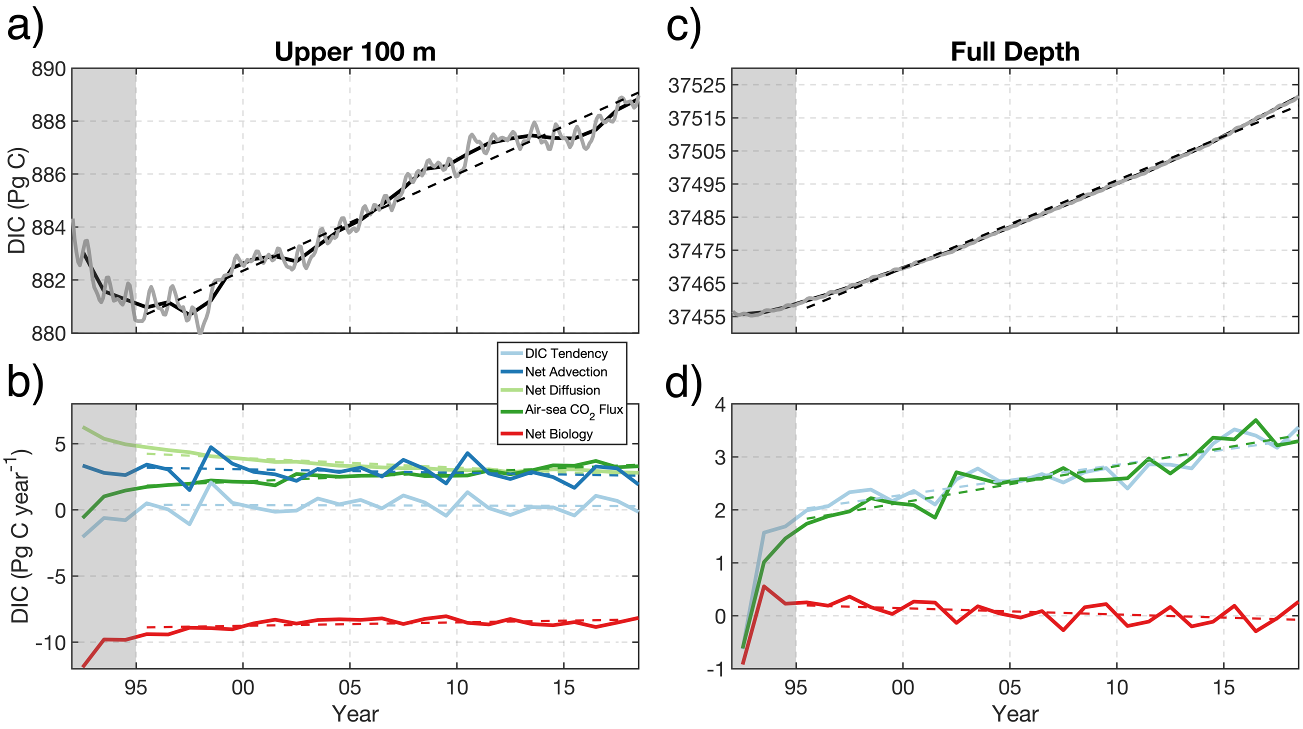

Figure S6. Same as Figure 9 in the main text except that, in the gray-shaded region, we show the 1992–1994 spin-up years, which have been discarded from the analysis. Note that a strong decrease in upper-100-m DIC is expected to occur in 1997 due to the strong El Niño conditions during that year.

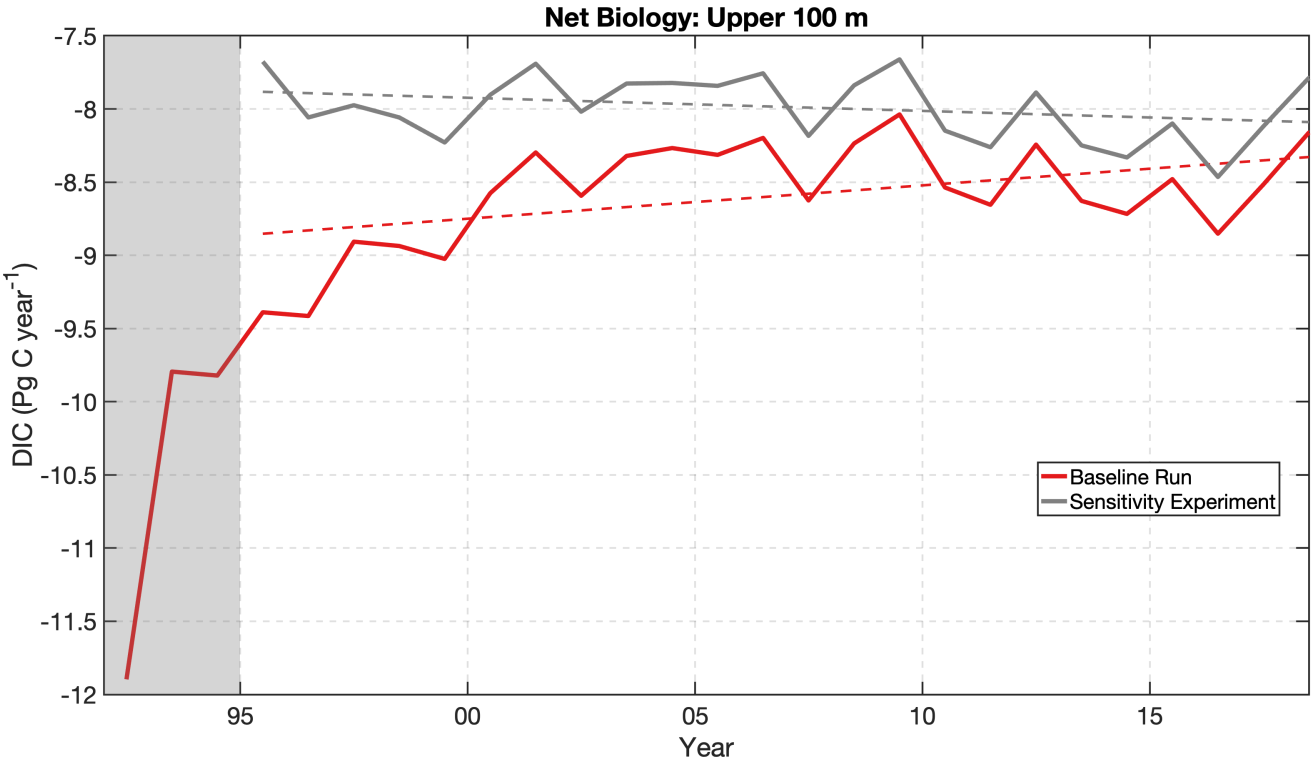

Figure S7. Time series of annual-mean net biology for the upper 100 m; red line shows the baseline run and black line shows the sensitivity experiment. Thin dashed lines show linear fits from 1995–2018; grey shading shows the model spin-up period. For the sensitivity experiment, the simulation starts in 1995 with initial DIC, NO_3, NO_2, NH_4, PO_4, Fe, and SiO_2 conditions taken from the baseline experiment at the end of 2018.

	DIC Tendency	Net Advection	Net Diffusion	Air-sea CO_2 Flux	Net Biology
**Global Ocean**	5.99E-04	4.16E-03	7.14E-03	9.27E-03	-2.00E-02
**All Biomes**	7.72E-04	3.39E-03	8.12E-03	9.82E-03	-2.06E-02
**sNH**	-3.04E-04	-1.13E-02	1.21E-02	2.48E-02	-2.59E-02
**sNS**	9.44E-04	3.23E-03	3.92E-03	1.73E-03	-7.93E-03
**sEQ**	1.03E-03	3.39E-02	4.99E-03	-7.82E-03	-3.00E-02
**sSS**	9.42E-04	4.36E-03	5.89E-03	2.05E-03	-1.14E-02
**sSH**	1.17E-03	6.63E-03	8.74E-03	1.05E-02	-2.47E-02
NP1	-9.93E-04	-8.84E-03	2.76E-03	1.62E-02	-1.12E-02
NP2	6.34E-05	-5.04E-03	1.48E-02	2.54E-02	-3.51E-02
NP3	8.24E-04	-1.76E-02	2.48E-02	3.03E-02	-3.67E-02
NP4	8.24E-04	2.57E-03	4.12E-03	3.28E-03	-9.15E-03
WPE	1.21E-03	1.33E-02	7.64E-03	-2.73E-03	-1.70E-02
EPE	1.16E-03	5.20E-02	3.20E-03	-1.39E-02	-4.01E-02
SP	9.48E-04	4.68E-03	6.15E-03	5.85E-04	-1.05E-02
NA1	-1.32E-03	-9.00E-03	8.57E-04	1.52E-02	-8.40E-03
NA2	-1.09E-04	-1.67E-02	1.60E-02	3.51E-02	-3.44E-02
NA3	7.15E-04	-1.48E-02	2.51E-02	2.24E-02	-3.20E-02
NA4	1.21E-03	4.70E-03	3.45E-03	-1.73E-03	-5.21E-03
AE	4.79E-04	2.93E-02	4.48E-03	-3.41E-03	-2.99E-02
SA	1.38E-03	7.14E-03	5.44E-03	9.81E-04	-1.22E-02
IO	7.02E-04	2.37E-03	5.70E-03	5.04E-03	-1.24E-02
SO1	9.35E-04	4.19E-03	1.67E-02	2.37E-02	-4.37E-02
SO2	1.35E-03	9.18E-03	1.17E-02	1.09E-02	-3.04E-02
SO3	1.14E-03	5.72E-03	2.16E-03	3.31E-03	-1.01E-02

Table S1. Amplitude of climatological seasonal cycle values for all budget terms shown in Figure 8a; units are in mol m^{-3} $year^{-1}$.

	DIC Tendency	Net Advection	Net Diffusion	Air-sea CO_2 Flux	Net Biology
**Global Ocean**	5.99E-04	4.16E-03	7.14E-03	9.27E-03	-2.00E-02
**All Biomes**	7.72E-04	3.39E-03	8.12E-03	9.82E-03	-2.06E-02
**sNH**	-3.04E-04	-1.13E-02	1.21E-02	2.48E-02	-2.59E-02
**sNS**	9.44E-04	3.23E-03	3.92E-03	1.73E-03	-7.93E-03
**sEQ**	1.03E-03	3.39E-02	4.99E-03	-7.82E-03	-3.00E-02
**sSS**	9.42E-04	4.36E-03	5.89E-03	2.05E-03	-1.14E-02
**sSH**	1.17E-03	6.63E-03	8.74E-03	1.05E-02	-2.47E-02
NP1	-9.93E-04	-8.84E-03	2.76E-03	1.62E-02	-1.12E-02
NP2	6.34E-05	-5.04E-03	1.48E-02	2.54E-02	-3.51E-02
NP3	8.24E-04	-1.76E-02	2.48E-02	3.03E-02	-3.67E-02
NP4	8.24E-04	2.57E-03	4.12E-03	3.28E-03	-9.15E-03
WPE	1.21E-03	1.33E-02	7.64E-03	-2.73E-03	-1.70E-02
EPE	1.16E-03	5.20E-02	3.20E-03	-1.39E-02	-4.01E-02
SP	9.48E-04	4.68E-03	6.15E-03	5.85E-04	-1.05E-02
NA1	-1.32E-03	-9.00E-03	8.57E-04	1.52E-02	-8.40E-03
NA2	-1.09E-04	-1.67E-02	1.60E-02	3.51E-02	-3.44E-02
NA3	7.15E-04	-1.48E-02	2.51E-02	2.24E-02	-3.20E-02
NA4	1.21E-03	4.70E-03	3.45E-03	-1.73E-03	-5.21E-03
AE	4.79E-04	2.93E-02	4.48E-03	-3.41E-03	-2.99E-02
SA	1.38E-03	7.14E-03	5.44E-03	9.81E-04	-1.22E-02
IO	7.02E-04	2.37E-03	5.70E-03	5.04E-03	-1.24E-02
SO1	9.35E-04	4.19E-03	1.67E-02	2.37E-02	-4.37E-02
SO2	1.35E-03	9.18E-03	1.17E-02	1.09E-02	-3.04E-02
SO3	1.14E-03	5.72E-03	2.16E-03	3.31E-03	-1.01E-02

Table S2. Time-mean values for all budget terms shown in Figure 11a; units are in mol m^{-3} $year^{-1}$.
